# Individual epigenetic status of the pathogenic D4Z4 macrosatellite correlates with disease in facioscapulohumeral muscular dystrophy

**DOI:** 10.1186/s13148-015-0072-6

**Published:** 2015-03-29

**Authors:** Takako I Jones, Oliver D King, Charis L Himeda, Sachiko Homma, Jennifer C J Chen, Mary Lou Beermann, Chi Yan, Charles P Emerson, Jeffrey B Miller, Kathryn R Wagner, Peter L Jones

**Affiliations:** Department of Neurology and Department of Cell and Developmental Biology, The Wellstone Program, University of Massachusetts Medical School, 55 Lake Avenue North, Worcester, MA 01655 USA; The Eunice Kennedy Shriver National Institute of Child Health and Human Development, Sen. Paul D. Wellstone Muscular Dystrophy Cooperative Research Center, 31 Center Drive, Bethesda, MD USA; Neuromuscular Biology & Disease Group, Departments of Neurology and Physiology & Biophysics, Boston University School of Medicine, 72 E Concord St, Boston, MA 02118 USA; Key Lab of Swine Genetics and Breeding, Ministry of Agriculture, College of Animal Science and Technology, Huazhong Agricultural University, No.1, Shizishan Street, Wuhan, 430070 People’s Republic of China; The Hugo W. Moser Research Institute, Kennedy Krieger Institute, and the Departments of Neurology and Neuroscience, The Johns Hopkins School of Medicine, 733 N Broadway, Baltimore, MD 21205 USA

**Keywords:** FSHD, Muscular dystrophy, DUX4, D4Z4, Epiallele, Epigenetic modifier, Disease modifier, Decitabine, DNA methylation

## Abstract

**Background:**

Both forms of facioscapulohumeral muscular dystrophy (FSHD) are associated with aberrant epigenetic regulation of the chromosome 4q35 D4Z4 macrosatellite. Chromatin changes due to large deletions of heterochromatin (FSHD1) or mutations in chromatin regulatory proteins (FSHD2) lead to relaxation of epigenetic repression and increased expression of the deleterious double homeobox 4 (*DUX4*) gene encoded within the distal D4Z4 repeat. However, many individuals with the genetic requirements for FSHD remain asymptomatic throughout their lives. Here we investigated family cohorts of FSHD1 individuals who were either affected (manifesting) or without any discernible weakness (nonmanifesting/asymptomatic) and their unaffected family members to determine if individual epigenetic status and stability of repression at the contracted 4q35 D4Z4 array in myocytes correlates with FSHD disease.

**Results:**

Family cohorts were analyzed for DNA methylation on the distal pathogenic 4q35 D4Z4 repeat on permissive A-type subtelomeres. We found DNA hypomethylation in FSHD1-affected subjects, hypermethylation in healthy controls, and distinctly intermediate levels of methylation in nonmanifesting subjects. We next tested if these differences in DNA methylation had functional relevance by assaying *DUX4-fl* expression and the stability of epigenetic repression of *DUX4-fl* in myogenic cells. Treatment with drugs that alter epigenetic status revealed that healthy cells were refractory to treatment, maintaining stable repression of *DUX4*, while FSHD1-affected cells were highly responsive to treatment and thus epigenetically poised to express *DUX4*. Myocytes from nonmanifesting subjects had significantly higher levels of DNA methylation and were more resistant to *DUX4* activation in response to epigenetic drug treatment than cells from FSHD1-affected first-degree relatives containing the same contraction, indicating that the epigenetic status of the contracted D4Z4 array is reflective of disease.

**Conclusions:**

The epigenetic status of the distal 4qA D4Z4 repeat correlates with FSHD disease; FSHD-affected subjects have hypomethylation, healthy unaffected subjects have hypermethylation, and nonmanifesting subjects have characteristically intermediate methylation. Thus, analysis of DNA methylation at the distal D4Z4 repeat could be used as a diagnostic indicator of developing clinical FSHD. In addition, the stability of epigenetic repression upstream of *DUX4* expression is a key regulator of disease and a viable therapeutic target.

**Electronic supplementary material:**

The online version of this article (doi:10.1186/s13148-015-0072-6) contains supplementary material, which is available to authorized users.

## Background

Facioscapulohumeral muscular dystrophy (FSHD) has long been characterized as an autosomal dominant genetic myopathy [1-3]; however, the critical role of epigenetic regulation in both forms of FSHD is now being recognized, and FSHD can accurately be characterized as an epigenetic disease [4-8]. FSHD is the most prevalent (affecting approximately 1:7,500 to 15,000 individuals) myopathy that indiscriminately afflicts children and adults of all ages and both genders [1,7,9,10]. FSHD1 (OMIM 158900) accounts for >95% of reported cases and results from a range of large DNA deletions within the 4q35 localized macrosatellite D4Z4 repeat array [11,12]. Healthy, genetically unaffected individuals are defined as having more than 10 D4Z4 repeat units (RUs) on both 4q chromosome arms (generally 25 to 35 RUs and as high as 120 RUs per array [13,14]), whereas individuals with genetic FSHD1 have between 1 and 10 D4Z4 RUs on one 4q chromosome arm, thus classifying FSHD as an autosomal dominant disease. These polymorphic FSHD1-sized D4Z4 contractions by themselves are not pathogenic, and development of FSHD also requires a disease-permissive allele of the chromosome 4q subtelomere (4A) in *cis* with the contracted array [14-17]. The far less common form, FSHD2 (OMIM 158901), presents with similar clinical features as FSHD1 but does not involve contraction of the D4Z4 array [4,18]. FSHD2 is, however, still genetically linked to the 4q35 region by the requirement of at least one permissive 4A-type subtelomere in order to develop disease [4,17].

**Figure 1:**
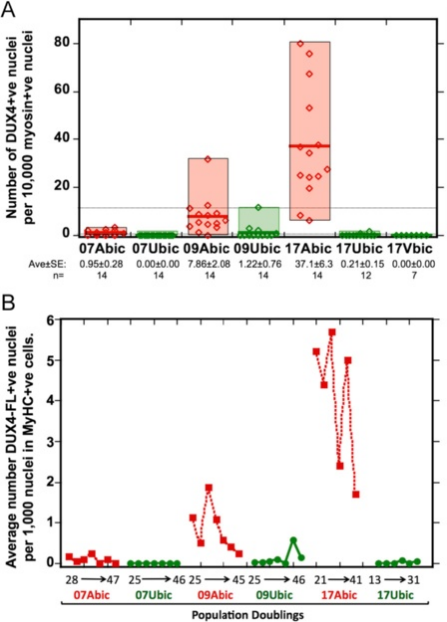
**Myogenic cells from different individuals have consistently different and stable frequencies of DUX4-FL expression. (A)** Myogenic cells from different individuals have different extents of DUX4-FL expression. DUX4-FL expression frequency (number of DUX4-FL-positive nuclei per 10,000 nuclei in myosin-positive cells) was measured in multiple independent cultures of differentiated myogenic cells from three FSHD patients (07Abic, 09Abic, and 17Abic; red) and their unaffected (control) family members (07Ubic, 09Ubic, 17Ubic, and 17Vbic, respectively; green). Within each family, FSHD cells had a significantly higher frequency of DUX4-FL expression than control cells (*P* < 0.01; *t*-tests; *n* = 12 to 14). DUX4-FL expression frequencies of cells from each FSHD patient also differed significantly from each other, with 17Abic > 09Abic > 07Abic (*P* < 0.01; *t*-tests; *n* = 12 to 14). Red open diamonds = FSHD; green open diamonds = unaffected control; horizontal bar = average; average ± SE and ‘*n*’ are shown below each culture name. **(B)** DUX4-FL expression frequency does not show a clear change upon serial subculture. Cultures of cells from the same FSHD and controls as in (A) were serially subcultured through 6-7 passages, and DUX4-FL expression frequency was measured at each passage in differentiated cultures as described in the ‘Methods.’ Each point (closed red squares for FSHD, closed green circles for controls) shows results for a single passage, with the passage number increasing from left to right in sequence. The beginning and ending number of total population doublings (PD) for each cell strain is shown below the name (e.g., for 07Abic, the cells were first examined at PD = 28 and these reached PD = 47 at the final passage examined).

Each of the D4Z4 RUs within the 4q35 macrosatellite contains 3.3 kb of highly GC-rich (73%) DNA, encompassing >16 nucleosomes, with multiple repeat sequences normally associated with heterochromatin [19]. Thus, FSHD1-sized deletions remove a substantial amount of regulatory heterochromatin from the 4q35 region, significantly altering the local epigenetic landscape of the contracted allele [20-22]. FSHD2 is also caused by the epigenetic disruption of the 4q35 D4Z4 array leading to aberrant gene expression; however, the dysregulation is not caused by the physical removal of regulatory heterochromatin as in FSHD1 but is due to mutations in gene(s) encoding the epigenetic machinery responsible for establishing and maintaining repression of the D4Z4 array [4,5]. More than 85% of FSHD2 cases analyzed to date are linked to mutations in the *SMCHD1* gene [5,23-25], which encodes a chromatin remodeling protein required for normal DNA methylation levels and transcriptional repression at certain loci, including D4Z4 arrays [26-28]. In addition, mutations in the *SMCHD1* gene increase the severity of FSHD1 [6,29], indicating that SMCHD1 is an epigenetic modifier of both forms of FSHD. Thus, epigenetic dysregulation of the 4q35 D4Z4 array, albeit through different mechanisms, links FSHD1 and FSHD2 [4,7,8].

A consequence of the epigenetic disruption at 4q35 in FSHD1 and FSHD2 is the increased expression and altered splicing of the double homeobox 4 (*DUX4*) gene to generate the *DUX4-fl* (*DUX4*-full length) mRNA in FSHD skeletal muscle, which results in aberrant expression of DUX4-FL and its downstream target genes with consequent pathology [17,30-36]. Although a copy of *DUX4* resides within each RU of the D4Z4 array [37], only *DUX4-fl* transcribed from the distal-most 4q35 D4Z4 repeat is stably expressed in FSHD due to the presence of a polyadenylation signal (PAS) in a permissive 4A subtelomere-specific exon distal to the array, which is absent in 4B and other non-permissive subtelomeres [17]. This distal third exon is spliced into the mRNA (thereby explaining the linkage of FSHD to the 4A-type subtelomeres) and translated to produce DUX4-FL protein [17,30]. However, DUX4-FL expression in FSHD is very low and shows cell-to-cell variability as <0.5% of the nuclei in FSHD1-derived myogenic cultures express DUX4-FL [30,33]. Although restricted to a small percentage of myonuclei at any one time, the aberrant expression of DUX4-FL is proposed to lead to progressive muscle atrophy and ultimately FSHD pathology [30-36,38-41]. Two studies have also reported expression of *DUX4-fl* mRNA and protein in some myogenic cells and muscle tissue from certain asymptomatic and healthy individuals [33,42], although at lower levels than in FSHD1 patients. Thus, *DUX4-fl* expression *per se* is not sufficient for developing clinical FSHD, suggesting the existence of disease modifiers both upstream and downstream of DUX4-FL.

As described above, one important class of disease modifier encompasses chromatin regulatory proteins, such as SMCHD1, that function to establish or maintain epigenetic repression of the D4Z4 array, thus affecting *DUX4-fl* expression. In addition, contracted D4Z4 arrays may be marked by different epigenetic states in different individuals due to shifts in the probabilistic establishment of these states during development, similar to the characteristics of metastable epialleles (reviewed in [43,44]). To investigate the role of epigenetic modifications in FSHD, we analyzed patterns of DNA methylation at the 4q35 D4Z4 array in family cohorts of myogenic cells from FSHD1-affected subjects, FSHD1-nonmanifesting carriers, and healthy controls. We determined that these cells have individual differences at the 4q35 D4Z4 array and these epigenetic differences affected the stability of *DUX4* silencing. The patterns of DNA methylation at the distal, pathogenic D4Z4 repeat, as well as inducibility of *DUX4-fl* expression following epigenetic drug treatment, correlated with disease manifestation and offer an explanation for how individuals can be genetically FSHD1 yet clinically asymptomatic.

## Results

There are several key distinguishing aspects of our analysis. We studied our well-characterized FSHD1 family cohorts of myogenic cells derived from muscle biopsies [33,45,46], thus minimizing differences related to genetic background and also allowing the analysis of multiple cohorts of FSHD1-affected subjects and nonmanifesting carriers containing the same D4Z4 contraction. FSHD is a myopathy, and *DUX4-fl* expression is induced in differentiated myogenic cells [47]; thus, the use of these cells, as opposed to the lymphocytes used in most other studies, allowed analysis of epigenetic status and pathogenic gene expression in the most affected cell type. In contrast to earlier studies which analyzed very few CpGs, our study used bisulfite sequencing (BSS), enabling analysis of the methylation status for >50 CpGs each in both the gene body and 5′ promoter region of *DUX4* [48]. Importantly, our BSS amplifications were specific to the 4qA D4Z4 (4qA and 4qA-L BSS assays) or the 4q and 10q D4Z4 RUs (DUX4 5′ BSS assay). Our assays did not amplify and assess the numerous D4Z4 homologs from other regions of the genome that are not associated with or epigenetically dysregulated in FSHD [48,49]. Finally, we specifically analyzed the pathogenic distal-most D4Z4 repeat for both DNA methylation status and stability of epigenetic repression as indicated by *DUX4-fl* expression. This is in contrast to most other studies which have analyzed four centromere-proximal D4Z4 repeats (two from 10q, one from the contracted 4q, and one from the non-contracted 4q); these studies do not specifically assess the pathogenic chromosome and they focus on a region far from the site of stable *DUX4-fl* expression [25]. Our unique approach provides the first epigenetic analysis of the distal *DUX4* gene associated with FSHD and identifies distinct epigenetic characteristics of healthy, FSHD1-affected, and FSHD1-nonmanifesting states.

### The frequency of DUX4-FL expression is stable in culture

Myogenic cells obtained from different individual donors have large differences in the frequency of DUX4-FL protein expression [33]. Therefore, we first determined if DUX4-FL levels in myogenic cells were stable upon repeated culturing. Our earlier study [33] raised the possibility that DUX4-FL expression frequencies differed depending on the donor; however, that study examined DUX4-FL protein in only one culture for most donors and did not determine if the number of population doublings affected DUX4-FL expression. In addition, DUX4-FL expression in myogenic cells is almost exclusive to differentiated myocytes, as identified by expression of myosin heavy chain (MyHC) [47]; our previous study reported the number of DUX4-FL-positive nuclei per 1,000 total nuclei in the cultures and thus did not account for possibly differing extents of differentiation among different cultures. Thus, to extend our previous study, we examined DUX4-FL expression frequencies at different population doublings (PD) using a serial subculturing assay (see the ‘Methods’ section) with differentiated FSHD and unaffected cells derived from the biceps or deltoid muscles of multiple individual donors (Additional file 1: Table S1). Upon repeated subculturing, we found that the doubling times of these primary cultures in growth medium began to slow by PD approximately 55 to 60, therefore we limited DUX4-FL expression experiments to differentiated cultures derived from myogenic cells at PD ≤ ~47, which was prior to the replicative limit.

Differentiated cells from three FSHD donors showed an almost 50 times difference in average frequency of DUX4-FL expression, with the frequency of DUX4-FL-positive nuclei per 1,000 nuclei in myosin-expressing cells ranging from approximately 0.1 (for 07Abic cultures) to approximately 4.7 (for 17Adel cultures) (Table 1). In addition, DUX4-FL expression frequencies were approximately equal for the biceps- and deltoid-derived cultures for each donor (Table 1). We noted that DUX4-FL expression frequencies in these three cohorts inversely correlated with D4Z4 array length as measured by *Eco*RI-*Bln*I restriction fragment length (Table 1 and Additional file 1: Table S1), which, despite the limited sample size, is potentially intriguing considering short 4q D4Z4 arrays (<5 RUs) are associated with severe FSHD disease while longer arrays show more inter-individual variation in clinical severity [20,25]. For these three FSHD donors, cultures of biceps-derived (Abic) and deltoid-derived (Adel) myogenic cells from 17A consistently had the highest frequencies of DUX4-FL expression, whereas cells from 09A typically had intermediate levels, and cells from 07A typically had the lowest level of DUX4-FL expression (Figure 1). Thus, FSHD cells obtained from different donors maintained consistently different frequencies of DUX4-FL expression upon repeated sub-culturing and over a range of total population doublings. For cells from each of three FSHD donors, the frequency of DUX4-FL-positive nuclei showed a weak trend to lower frequency of expression at higher passages and population doublings (*R*^2^ = 0.16 for 07Abic, 0.32 for 09Abic, and 0.39 for 17Abic).

**Table 1:** **DUX4-FL expression in differentiated myogenic cell cultures by individual donor and muscle of origin**

**Family**	**Donor**	***Eco*** **RI/** ***Bln*** **I fragment sizes**	**Disease status**	**#DUX4-FL + nuclei per 1,000 nuclei in MyHC + cells (average ± SE (** ***n*** **))**	
				**Biceps-derived (bic)**	**Deltoid-derived (del)**
07	07A	29 kb 4A161	FSHD manifesting	0.095 ± 0.028 (14)**	0.17 ± 0.09 (4)
		53 kb 4A161			
	07U	34 kb 4B163	Unaffected	0.00 ± 0.00 (14)**	0.015 ± 0.015 (4)
		53 kb 4A161			
09	09A	25 kb 4A161	FSHD manifesting	0.79 ± 0.21 (14)**	2.14 ± 0.84 (4)*
		>112 kb 4B168			
	09U	>112 kb 4A161	Unaffected	0.12 ± 0.08 (14)**	0.00 ± 0.00 (4)*
		>112 kb 4A166H			
17	17A	19 kb 4A161	FSHD manifesting	3.71 ± 0.63 (14)**	4.76 ± 0.97 (4)**
		87 kb 4A-L161			
	17U	97 kb 4B163	Unaffected	0.021 ± 0.015 (12)**	0.012 ± 0.012 (4)**
		>112 kb 4A161			
	17 V	90 kb 4A161	Unaffected	0.00 ± 0.00 (7)**	n.d.
		>112 kb 4B168			

*P < 0.05, **P < 0.01 by *t*-test for FSHD vs. unaffected within the indicated family. n.d. = not done.

Consistent with our earlier work [33], we also detected a low frequency of DUX4-FL expression in nuclei within differentiated (MyHC-positive) cells from two of the four healthy (non-FSHD) donors (Table 1). Cells from these two unaffected donors showed a weak trend to higher DUX4-FL expression after repeated subculturing (*R*^2^ = 0.31 for 09Ubic and 0.26 for 17Ubic). As with our previous study investigating DUX4-FL expression in large single cultures of myogenic cells from nine of the Wellstone Center cohorts (03, 07, 09, 12, 15, 16, 17, 18, 20) [33], for each of the three donor families (07, 09, 17), the average frequency of DUX4-FL-expressing nuclei was higher in differentiated cells from the FSHD donor than from the unaffected donor across multiple cultures (Table 1, *n* = 4 to 14); this difference reached significance (*P* < 0.05, *t*-test) in every case except 07Adel *vs.* 07Udel (*P* < 0.15) (Table 1). Thus, the percentage of myonuclei that expressed DUX4-FL varied among cell cultures isolated from different individuals but remained relatively stable among different cultures derived from the same donor biopsy. In cultures from all individuals tested, derived from 13 different biopsies, the number of DUX4-FL expressing nuclei remained stable upon repeated subculturing, indicating that the mechanisms regulating DUX4-FL expression are similarly stable in myocyte cell culture.

### Myogenic cells derived from FSHD1-affected subjects are significantly hypomethylated at the distal D4Z4 unit of a contracted 4q array compared with the non-contracted allele and healthy controls

**Figure 2:**
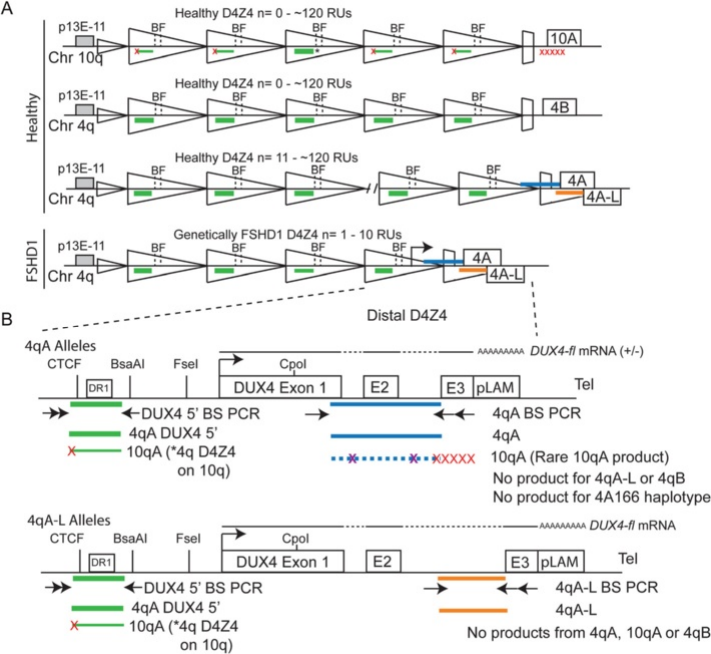
**Specific DNA methylation analysis of the FSHD-associated distal 4q D4Z4 repeat unit on A-type subtelomeres by BSS sequencing. (A)** Schematic representations of D4Z4 arrays on 4q and 10q chromosomes. Healthy unaffected individuals have any combination of two of the non-contracted 4q chromosomes; FSHD1 is not associated with 4qB or 10qA chromosomes. FSHD1-affected and nonmanifesting subjects have at least one contracted 4qA array and are distinguished clinically by disease presentation. The regions assayed by BSS are indicated as follows: 4qA BSS assay (blue bars), 4qA-L BSS assay (orange bars), and DUX4 5′ BSS assay (green bars). B = *Bsa*AI and F = *Fse*I restriction sites often used for DNA methylation analysis. **(B)** Schematic of the distal 4qA (upper) and 4qA-L (lower) D4Z4 RUs analyzed in this study. Black arrows indicate PCR primer locations and red Xs indicate five sequence changes between 4A and 10A within the primers. Rare 10qA products can be amplified in the 4qA BSS assay if PCR primers degrade; however, these are clearly identified by sequence polymorphisms (purple Xs) and removed from analysis.

Overall DNA methylation levels of the 4q35 D4Z4 repeat array differ significantly between healthy cells, which are hypermethylated (>50% methylation of assayed restriction enzyme sites) on both 4q alleles, and cells derived from FSHD1-affected subjects, which are comparatively hypomethylated (<35% methylation of assayed restriction enzyme sites) on the contracted 4q allele [4,20,50]. While an earlier study found no significant correlation between disease severity and methylation among FSHD1-affected subjects [20], it did suggest that hypomethylation may, like disease severity, be more pronounced for those subjects with shorter D4Z4 arrays. As mutations in the chromatin regulator *SMCHD1* can increase clinical severity in FSHD1 families [6,29], it is likely that the overall epigenetic state of the 4q35 D4Z4 array can affect the clinical phenotype, even when taking D4Z4 array length into account. Of note, previous reports on FSHD1 DNA methylation assayed only a few CpGs in methylation-sensitive restriction sites either in rare genotypes [20,50] or in a combined analysis of the most centromeric D4Z4 repeat of both 4q and 10q chromosomes as a proxy for the epigenetic status of the array [4,25], or analyzed all 4q and 10q D4Z4 RUs as a group (Figure 2) [51]. In particular, one recent epigenetic study did not distinguish the contracted chromosome from the three other non-pathogenic chromosomes [51]. Another study used global estimates of methylation as a function of D4Z4 repeat lengths to detect deviation from predicted average methylation [25], which requires complete knowledge of genotype and cannot ascribe deviation from the predictions to any particular allele. Regardless of the chosen method, all previous studies failed to capture the epigenetic status of the pathogenic distal D4Z4 repeat on the contracted FSHD1 allele [4,20,25,50-52], which may differ between genetically FSHD1 individuals. Considering the stable differences in the number of DUX4-FL expressing myonuclei among cultures from FSHD1-affected subjects, we therefore investigated the DNA methylation profiles of the distal D4Z4 repeat on healthy and FSHD1 alleles and assessed the stability of epigenetic repression in myocytes at the 4q35 D4Z4 array using *DUX4-fl* mRNA expression as a read-out for chromatin relaxation. To further address potential connections to FSHD1 disease severity, without the confounding effects of 4qA contraction length or haplotype, we also analyzed familial nonmanifesting carriers of FSHD1-sized contractions.

**Figure 3:**
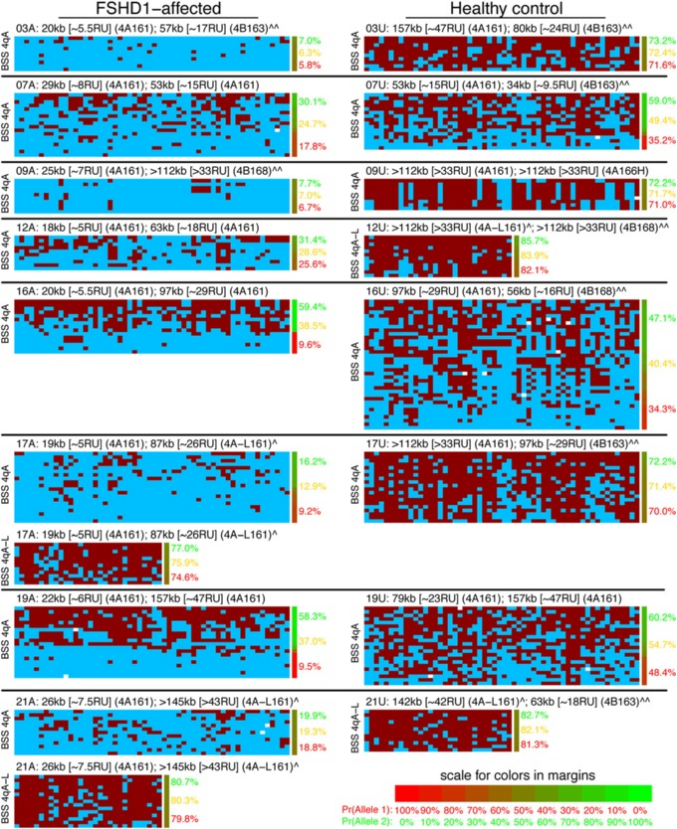
**DNA methylation levels in myocytes at the distal D4Z4 repeat on the contracted 4qA chromosome correlate with disease.** BSS analysis of the distal pathogenic D4Z4 RU in family cohorts of myogenic cells derived from biceps (cohorts 03, 07, 09, 12, 16, 17, 19, and 21) of FSHD1-affected (left column) and unaffected (right column) subjects. Overall, 56 predicted CpGs (each represented as a column, numbered 1-56, left to right) arranged linearly on a chromosome were assayed for the 4qA BSS assay, or 30 predicted CpGs (columns numbered 1-30, left to right) for the 4qA-L BSS assay, as indicated in the left margin. Each independent chromosome assayed is represented by a row with each CpG represented by a box (red boxes indicating methylation, blue boxes indicating lack of methylation, and empty boxes indicating lack of a CpG detected at that site). Importantly, on average >99% of the predicted CpGs were identified in the sequences analyzed for each sample, and each total sequence had >98% identity to the reference sequence, indicating that the amplified BSS products are specific to 4qA and there are very few polymorphisms. The haplotypes, associated *Eco*RI/*Bln*I fragment sizes, and calculated D4Z4 RUs of the shortest FSHD-permissive allele are listed after sample names taken from Additional file 1: Table S1; symbols ^ and ^^ are described there. Numbers in the right margin indicate estimated percent methylation for each of two alleles using a beta-binomial mixture model (allele 1 in red, allele 2 in green), and using a mono-allelic model (orange). The color bar in the right margin indicates confidence in assignment of each sequence to each allele: pure red for 100% posterior probability for allele 1; pure green for 100% posterior probability for allele 2; blended colors for intermediate values (color scale in the lower right).

**Figure 4:**
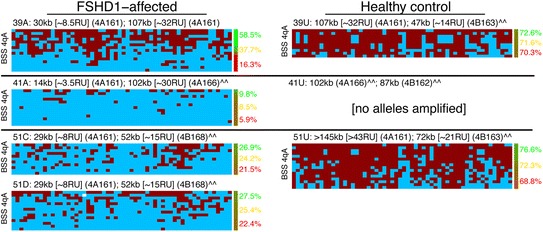
**DNA methylation levels in PBMCs at the distal D4Z4 repeat on the contracted 4qA chromosome correlate with disease.** BSS analysis (as described in Figure 3) of the distal pathogenic D4Z4 RU in family cohorts of PBMCs derived from blood of FSHD1-affected (left column) and healthy unaffected (right column) subjects. Refer to the Figure 3 legend for additional details and descriptions.

**Figure 5:**
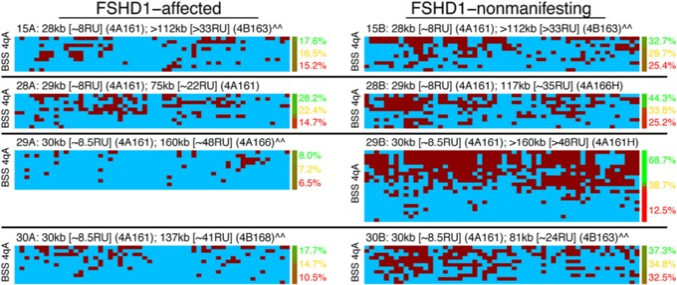
**FSHD1-affected subjects have lower DNA methylation levels in myocytes at the distal pathogenic D4Z4 repeat than nonmanifesting relatives.** BSS analysis (as described in Figure 3) of the distal pathogenic D4Z4 RU in family cohorts of myogenic cells from FSHD1-affected (left column) and FSHD1-nonmanifesting (right column) subjects. As in Figure 3, on average >99% of the predicted CpGs for the 4qA D4Z4 region were identified in the sequences analyzed for each sample, indicating that the amplified BSS products are specific to 4qA. Refer to the Figure 3 legend for additional details and descriptions.

**Figure 6:**
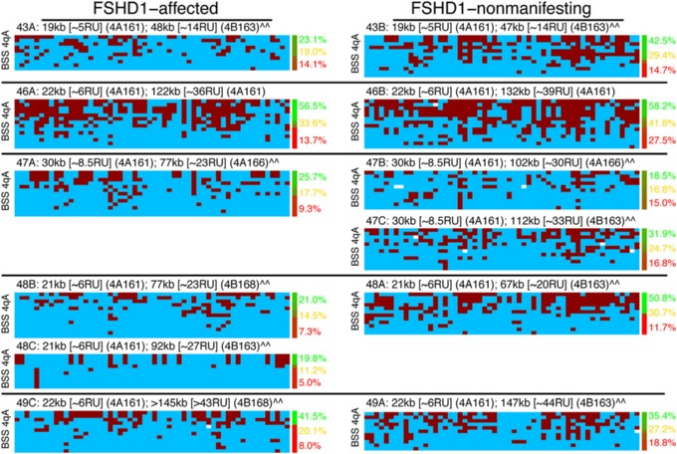
**FSHD1-affected subjects have lower DNA methylation levels in PBMCs at the distal pathogenic D4Z4 repeat than nonmanifesting relatives.** BSS analysis (as described in Figure 3) of the distal pathogenic D4Z4 RU in family cohorts of PBMCs from FSHD1-affected (left column) and FSHD1-nonmanifesting (right column) subjects. Refer to the Figure 3 legend for additional details and descriptions.

We developed two BSS assays specific for analyzing the DNA methylation status of the distal D4Z4 on 4qA chromosomes (Figure 2 and [48]) by utilizing polymorphisms in the primers that are exclusive to 4A and not found in 10A or 4B [17]. The 4qA BSS assay analyzes 56 CpGs in the distal D4Z4 RU on 4qA-containing chromosomes, as diagrammed in Figure 2B. A fraction of chromosomes characterized as 4qA are actually an allelic variant termed 4qA-L; these contain an additional 2 kb of D4Z4 sequence at the distal repeat, resulting in a much larger *DUX4* intron 2, while the distal exon 3 and A-type subtelomere are unchanged. Thus, the 4qA-L BSS assay utilizes the same 4A-specific reverse BS-PCR primers as the 4qA assay but analyzes a distinct set of 30 CpGs in the distal repeat on 4qA-L chromosomes. For comparisons with our 4qA and 4qA-L BSS analyses, as well as with other published studies [51,52], we designed a BSS analysis upstream of the *DUX4* open reading frame (DUX4 5′ BSS assay, Figure 2), which analyzes the methylation status of 59 CpGs. This DUX4 5′ region is amplified exclusively from all 4q/10q-type D4Z4 RUs, not from other D4Z4 homologs [49], and encompasses a putative CTCF binding site and the DR1 region that is hypomethylated in all 4q/10q D4Z4 RUs in FSHD2 cells [52,53]. It was critically important that we found these BSS assays to be specific to 4q (4qA and 4qA-L BSS assays) or 4q/10q D4Z4s (DUX4 5′ BSS assay), as indicated by the >99.8% coverage of expected CpGs when compared to the reference sequences (Figures 3, 4, 5, and 6; Additional file 1: Figures S1 and S2), because there are D4Z4 homologs on chromosomes 3, 13, 14, 15, 21, 22, and Y which do not show epigenetic changes in FSHD [49]. Fortunately, the 4q and 10q D4Z4s have very high sequence conservation and very few polymorphisms, so even if occasional non-4q/10q D4Z4s were amplified, they would be readily distinguished by their high degree of sequence polymorphisms and discarded from analysis [49]. Thus, combining the 4qA/4qA-L BSS and DUX4 5′ BSS provides a specific and detailed analysis of DNA methylation patterns at the pathogenic distal 4qA D4Z4 in the context of overall 4q/10q D4Z4 DNA methylation in FSHD1-affected, nonmanifesting, and healthy control cells.

We used the BSS assays described above to compare DNA methylation profiles (Figure 3) in myogenic cells from eight familial cohorts (03, 07, 09, 12, 16, 17, 19, and 21) representing clinically affected (manifesting) FSHD1 subjects that showed low (03A, 07A), mid-level (09A), and high (17A) percentages of DUX4-FL expressing myonuclei, and healthy controls (U). In addition, we assayed peripheral blood mononuclear cells (PBMCs) from three familial cohorts (39, 41, and 51) (Figure 4). In subjects with only one 4qA allele (Additional file 1: Table S1), all of the 4qA BSS data was derived from a single allele. Similarly, in subjects with one 4qA-L allele (Additional file 1: Table S1), all of the 4qA-L BSS data was derived from a single allele. In subjects with two 4qA alleles, 50% of the BSS sequences are expected to originate from each of the two 4q alleles (although the precise percent may differ due to random sampling fluctuations). Thus, for FSHD1 subjects, 50% of the sequences are expected to originate from the pathogenic D4Z4 RU and 50% from the non-contracted distal D4Z4 RU. However, to prevent high and variable methylation at the non-contracted allele from masking or diluting the signal from the contracted allele, we used a statistical mixture-model to estimate the average percent methylation for just the least-methylated of the 4qA or 4qA-L alleles (see the ‘Methods’ section). As expected, the cells from unaffected subjects were hypermethylated (on average 71% methylation across the region for myocytes, 62% for PBMCs) and the cells from 11 FSHD1-affected subjects were hypomethylated (on average 7% for myocytes, 14% for PBMCs). However, despite a >50-fold range in DUX4-FL expressing myonuclei between the FSHD1 samples (Figure 1 and [33]), there were only small differences in average 4qA DNA methylation (03A = 5.8%, 07A = 17.8%, 09A = 6.7%, and 17A = 9.2%) for the contracted 4qA chromosomes analyzed for each subject. BSS analysis of the DUX4 5′ region supported these results (Additional file 1: Figure S1). Cells from FSHD1-affected subjects displayed higher overall average methylation at the DUX4 5′ region than at the 4qA region, but this is to be expected because the non-contracted 4q and both 10q chromosomes are included in analysis of the 5′ region; moreover, since any D4Z4 repeat (not just the distal-most) may be amplified in this PCR assay, the contracted 4qA allele makes a proportionately smaller contribution to the overall methylation.

Overall, in cells from FSHD1-affected subjects, the contracted 4qA allele is specifically hypomethylated and the non-contracted allele remains hypermethylated. DNA methylation levels at the distal D4Z4 unit are dramatically higher for healthy than for FSHD1-affected cells (*P* = 2 × 10^−12^, likelihood ratio test (LRT)), correlating with the correspondingly lower numbers of DUX4-FL expressing myonuclei in healthy cells. However, DNA methylation levels alone do not explain differences in the number of DUX4-FL expressing myonuclei among cells from different FSHD1-affected subjects or explain why so few FSHD1-affected myonuclei in a culture express DUX4-FL. Since DNA methylation is only one component of the epigenetic regulation, it is likely that there are additional differences in the overall chromatin state that can account for these changes in expression levels and frequency.

### Myogenic cells from FSHD1-nonmanifesting subjects have intermediate DNA methylation levels at the distal *DUX4* on the contracted 4q allele

The existence of nonmanifesting carriers of FSHD1-sized 4q35 D4Z4 arrays in FSHD1-affected families has been known for many years, and more recently, a high prevalence of D4Z4 array contractions with FSHD-permissive alleles in the general healthy population has been reported [33,54-60]. Considering that the 4q35 epigenetic status is dramatically different between FSHD1-affected and healthy subjects, we hypothesized that these differences could account for the different disease outcomes between FSHD1 subjects and relatives possessing the same genetic deletion but varying manifestations of weakness. Therefore, nine family cohorts of genetic FSHD1 subjects with manifesting and nonmanifesting members (Additional file 1: Table S1) [33] were profiled with the 4qA/A-L BSS analysis, four using myogenic cells and five using PBMCs (Figures 5 and 6) [33]. Within each family, myocytes from the nonmanifesting subject(s) had higher estimated D4Z4 DNA methylation arising from the contracted allele than myocytes from the manifesting subject(s) (Table 2). DNA methylation analysis of the DUX4 5′ region for four of the cohorts revealed a similar trend upstream of the gene body with higher average levels of DNA methylation for each nonmanifesting subject compared with the familial manifesting subject (Additional file 1: Figure S2). Thus, despite having the same FSHD1-sized allele, cells from nonmanifesting individuals had higher DNA methylation levels compared with those of manifesting subjects in both the pathogenic distal DUX4 gene body and the DUX4 promoter regions. In every case, nonmanifesting individuals were about the same age or much older than their manifesting relative (Additional file 1: Table S1), indicating that increased age does not correlate with loss of methylation.

**Table 2:** **Comparison of percent DNA methylation between cells derived from FSHD1-affected and nonmanifesting familial cohorts**

**Cohort**	**Manifesting (%)**	**Nonmanifesting (%)**	***Eco*** **RI/** ***Bln*** **I (kb)**	D4Z4 RU*
15	15.2	25.4	28	8
28	14.6	25.2	29	8
29	6.5	12.5	30	8.5
30	10.6	32.6	30	8.5
43	14.2	15.5	19	5
46	13.7	27.6	22	6
47	9.3	14.9 and 16.9	30	8.5
48	7.3 and 4.9	11.7	21	6
49	8.0	18.8	22	6

Comparison of percent DNA methylation using the 4qA BSS assay. *Calculated as D4Z4 RU = (*Eco*RI/*Bln*I fragment kb − 2 kb)/3.3 [15].

In summary, higher DNA methylation levels at the distal 4q35 D4Z4 unit on the contracted 4qA allele were significantly correlated with decreased FSHD disease severity in individuals who shared the same FSHD1 deletion (*P* = 0.004 by a nonparametric sign test, for any choice of which subject to include for the two cases of two affected or two nonmanifesting subjects in a family). This increased level of DNA methylation in nonmanifesting *vs.* manifesting subjects was also significant in a parametric linear mixed-effects analysis (see the ‘Methods’ section), in which levels for nonmanifesting carriers of FSHD1 contractions are slightly but significantly higher than for manifesting subjects (*P* = 0.02, LRT), but significantly lower than for healthy controls (*P* = 1 × 10^−7^, LRT). Notably, there was no significant difference between myogenic cells and blood cells (*P* = 0.53, LRT), which makes blood samples appealing as a less-invasive alternative to muscle biopsies, at least for studies of DUX4 methylation.

We conclude that, with respect to the pathogenic distal D4Z4 repeat on the contracted 4qA allele (when appropriate), healthy subjects display DNA hypermethylation, FSHD1 subjects manifesting weakness display hypomethylation, and FSHD1-nonmanifesting subjects display intermediate levels of methylation, slightly but significantly higher than those of FSHD1-affected subjects.

### Stability of epigenetic repression is variable between myogenic cells derived from FSHD1-affected and nonmanifesting subjects

**Figure 7:**
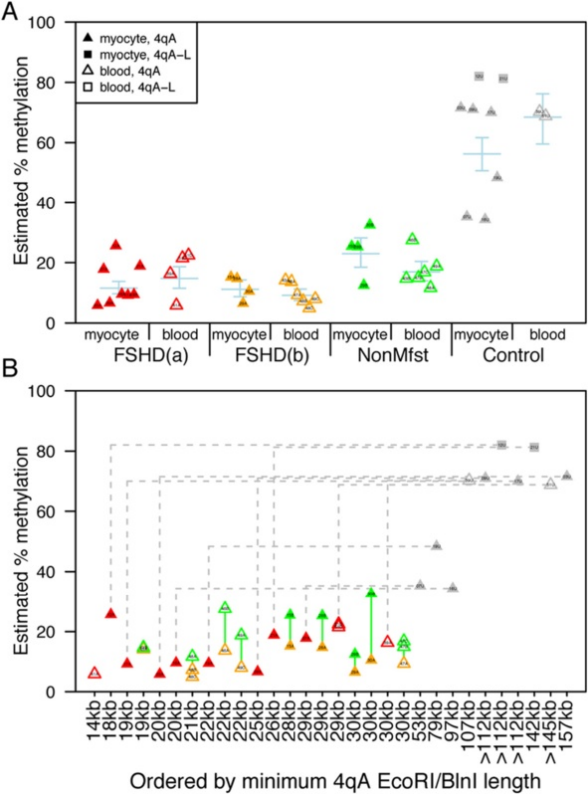
**Summary of DNA methylation data. (A)** A plot of the DUX4 gene body estimated average percent methylation for each sample, using a mixture-model to estimate this value for the 4qA allele with the lesser percent. FSHD-affected samples are split into two groups, those with nonmanifesting first-degree relatives in the sample cohort (FSHD(b); orange) and those without (FSHD(a); red). The nonmanifesting samples are labeled NonMfst (green), and the unaffected control samples are labeled Control (grey). Solid symbols indicate myocyte samples, and empty symbols indicate blood samples. Triangles indicate data from the 4qA assay and squares indicate data from the 4qA-L assay. Each group is subdivided into myocyte and blood subgroups. Within each of the eight subgroups, symbols are ordered by family number. Blue crosses behind each subgroup indicate means ± standard errors based on a linear mixed effect (LME) model with fixed effects for each of these eight subgroups, an additive fixed effect for assay type, and a random effect for family. Means and error bars show estimated fixed effects for 4qA assay; 4qA-L estimates are higher. LME calculations were performed on logit-transformed methylation probabilities, and results were then transformed back to percentages using a logistic transformation (which is why the error bars are not symmetric about the means). **(B)** The same data as in (A), with the same color scheme, but with samples ordered by minimum 4qA *Eco*RI/*Bln*I length (ordered by family number in case of ties) and with lines connecting related subjects. Vertical green lines connect FSHD-affected and nonmanifesting pairs (who have the same minimum 4qA *Eco*RI/*Bln*I length), and dashed grey lines connect FSHD-affected and control pairs (who do not). Vertical green line is not visible for family 43 (fourth column).

**Figure 8:**
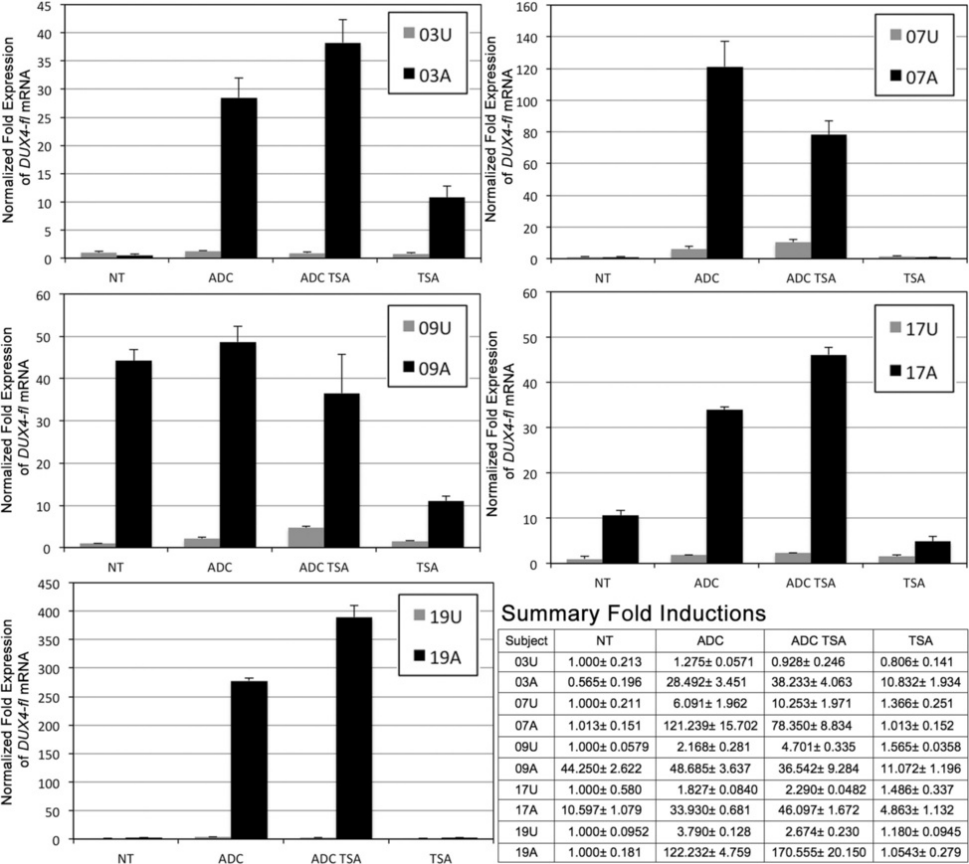
**Myocytes from FSHD1-affected subjects are epigenetically poised to express** ***DUX4-fl*** **.** Myocytes from five family cohorts (03, 07, 09, 17, and 19) of clinically affected FSHD1 subjects (A) and healthy first-degree relative controls (U) were treated in parallel with Decitabine (ADC), TSA, ADC + TSA (ADC TSA), or left untreated (NT). *DUX4-fl* expression was analyzed by qRT-PCR and normalized to levels of *18S* RNA. Data are plotted as fold expression relative to the untreated control sample for each cohort and summarized in the table, lower right. All assays were repeated three times and each qRT-PCR was performed in triplicate.

**Figure 9:**
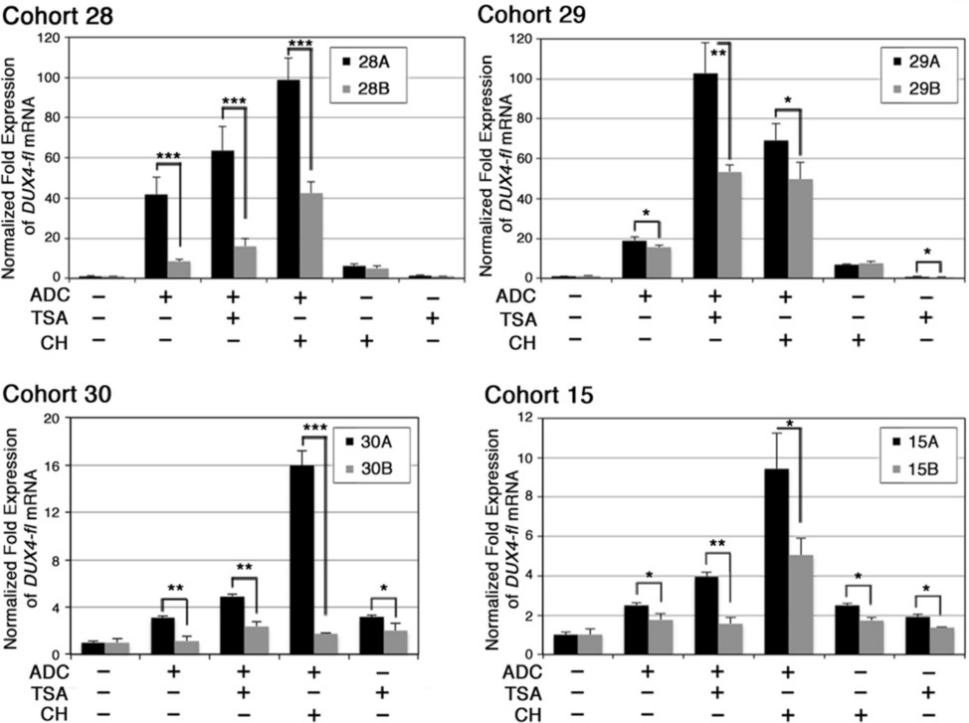
**Myocytes from FSHD1-nonmanifesting subjects are more refractory to expressing** ***DUX4-fl*** **than myocytes from FSHD1-affected relatives.** Myocytes from four family cohorts (15, 28, 29, and 30) of FSHD1-affected subjects (black bars, ‘A’ subjects) and FSHD1-nonmanifesting subjects (gray bars, ‘B’ subjects) were treated in parallel with Decitabine (ADC), TSA, chaetocin (CH), or combinations of drug treatments, as indicated. *DUX4-fl* expression was analyzed by qRT-PCR, normalized to levels of 18S RNA, and plotted as fold expression compared to the untreated samples for each cell strain. Comparisons were between FSHD1-affected and FSHD1-nonmanifesting for each treatment (*P < 0.05; **P < 0.01, ***P < 0.001, Student’s *t*-test). All assays were repeated three times and each qRT-PCR was performed in triplicate.

In myogenic cell cultures, cells from FSHD1-affected subjects have a very small percentage of nuclei (1:300 to 1:10,000) that express detectable levels of DUX4-FL protein (Figure 1), and levels of *DUX4-fl* mRNA are extremely low [30,33]. However, virtually all D4Z4-contracted chromosomes analyzed from FSHD1-affected subjects showed robust DNA hypomethylation (Figures 3, 4, 5, 6, and 7), indicating that epigenetic repression of *DUX4* expression (or stability) is still maintained in the vast majority of myonuclei. Since chromatin states are complex and DNA methylation levels are only one indication of the local chromatin environment, we asked if there were differences in the stability of D4Z4 repression in our family cohorts. To interrogate the epigenetic repression of the 4q35 D4Z4 arrays, cultures of myogenic cells were treated with 5-aza-2′-deoxycytidine (Decitabine/ADC) [61] and/or Trichostatin A (TSA) [62] and *DUX4-fl* mRNA expression was assayed by qRT-PCR (Figures 8 and 9). Decitabine treatment directly leads to decreases in DNA methylation levels [61,63] and, at certain loci, indirectly causes the reduction of repressive histone marks and the establishment of a permissive chromatin environment marked by nucleosome depletion and histone acetylation [64-66]. TSA is a broad-spectrum histone deacetylase (HDAC) inhibitor that can alter chromatin content by blocking the removal of acetyl groups from histones (and other acetylated non-histone targets) and inhibiting recruitment of some heterochromatin proteins [62,67,68]. Treatment with either Decitabine or TSA relieves epigenetic repression of certain loci, leading to gene activation [69,70], and the combination of the two drugs can have a synergistic effect [71]. We tested whether treatment with these small molecule enzyme inhibitors might decrease the repressive chromatin content of the D4Z4 array and potentially affect *DUX4-fl* expression levels.

As seen previously for DUX4-FL protein expression (Figure 1), initial *DUX4-fl* mRNA levels for the five cohorts analyzed were variable among the FSHD1 cells, while healthy control cells expressed *DUX4-fl* at much lower levels. FSHD1-affected and control cells were treated with Decitabine, TSA, or both, and *DUX4-fl* expression was assayed by qRT-PCR (Figure 8). *DUX4-fl* was detected in FSHD1-affected cells from both cohorts and, at much lower levels, in healthy controls, consistent with our previous study [33]. Surprisingly, Decitabine treatment of healthy cells, which are hypermethylated at the 4q35 D4Z4 array, only mildly induced *DUX4-fl* levels and the absolute levels never approached those found in Decitabine-treated cells from FSHD1-affected subjects (Figure 8). Similarly, treatment with TSA had no effect on *DUX4-fl* levels in any of the healthy controls. Interestingly, the combination of Decitabine and TSA treatment had a small effect on induction in two of the five healthy lines (09U, 4.7-fold; 07U, 10.2-fold); however, again, the resulting *DUX4-fl* levels were well below those of the treated cells from all five FSHD-affected subjects (Figure 8). To control for efficacy of drug treatment, we assayed the expression of the ankyrin repeat domain 1 (*ANKRD1*) gene, which is known to be epigenetically regulated in myocytes [72], and found that Decitabine/TSA treatment significantly induced its expression equally in both unaffected and affected cells (Additional file 1: Figure S6). Thus, with respect to *DUX4-fl* expression, healthy control cells are refractory to these epigenetic drug treatments, suggesting that normal repression of the non-contracted D4Z4 array is very stable.

Conversely, Decitabine treatment of FSHD1-affected cells, which are already hypomethylated compared with controls at the distal D4Z4 RU (Figures 3 and 4), significantly induced *DUX4-fl* in four of the five subjects (03A, 50-fold; 07A, 120-fold; 17A, 3.2-fold; 19A, 122-fold) with three of the five showing >50-fold induction. The lone cell line (09A) that did not show induction by Decitabine had the highest levels of *DUX4-fl* mRNA in the untreated sample, and >40-fold more than its corresponding control cell line (09U), suggesting that these cells may have already reached the maximum level of epigenetic relaxation attainable. Of the five cohorts, only 03A, which expressed the lowest levels of *DUX4-fl* of all the untreated FSHD-affected cells, showed induction by TSA alone. We conclude that myogenic cells from FSHD1-affected subjects have less stable epigenetic repression of *DUX4* than their healthy counterparts, and although the majority of cells do not express *DUX4-fl*, they are epigenetically poised for *DUX4-fl* expression.

Similarly, four family cohorts of myogenic cells from FSHD1-affected and nonmanifesting subjects were assayed for their response to Decitabine and/or TSA treatment (Figure 9). Again, Decitabine induced *DUX4-fl* expression in cells from FSHD1 individuals manifesting weakness in all four cohorts (15A, 28A, 29A, and 30A), while TSA alone had little to no effect. For 29A, the combination of Decitabine and TSA synergistically induced *DUX4-fl* expression. In parallel, cells from familial nonmanifesting subjects were subjected to the same set of drug treatments and assayed for *DUX4-fl* expression. For cells from nonmanifesting subject 29B, the pattern of induction was similar, although less pronounced, to that for cells from FSHD1 subject 29A. However, cells from nonmanifesting subjects 15B, 28B, and 30B showed little to no response to Decitabine or TSA, either alone or in combination.

In addition to FSHD-dependent changes in DNA methylation and histone acetylation states, changes in histone methylation at the FSHD locus have also been reported. These changes include reduced histone H3 lysine 9 tri-methylation (H3K9me3) and loss of its binding protein, heterochromatin protein 1 (HP1) [21,49]. Reducing the levels of H3K9me3 with chaetocin (CH), an inhibitor of the SUV39H1 methyltransferase responsible for establishing H3K9me3, induces *DUX4-fl* expression in immortalized human KD3 myoblasts [49,73,74]. Therefore, we assessed *DUX4-fl* induction by CH in these cohorts of FSHD-affected and nonmanifesting cells (Figure 9). Similar to treatment with Decitabine, treatment with CH alone induced *DUX4-fl* expression, and the combination of both increased expression even further. Again, for each treatment, cells from FSHD1 subjects manifesting weakness had higher *DUX4-fl* levels than cells from their nonmanifesting relatives with the identical 4qA allele. Thus, the repression of *DUX4-fl* in cells from nonmanifesting carriers is more refractory to induction by epigenetic drugs than in cells from their clinically affected relatives, despite sharing the same D4Z4 contraction.

## Discussion

### Patterns of DNA methylation at the pathogenic D4Z4 correlate with disease outcome in FSHD and can distinguish between FSHD1-affected, FSHD1-nonmanifesting, and healthy controls

Studies investigating FSHD1 families have identified asymptomatic individuals that share the same FSHD1 genetic diagnosis as their affected relatives yet report no noticeable muscle weakness [25,33,54,56-58]. Similarly, larger studies of normal individuals with no known FSHD relatives revealed that there are many individuals - reportedly approximately 1% to 3% of certain populations - that fit the current FSHD1 genetic diagnostic criteria yet show no clinical manifestation of the disease [60,75]. It is established that the overall epigenetic status of the 4q35 D4Z4 macrosatellite is distinctly altered between FSHD-affected and healthy control subjects [4,20,21,49,50,76]. Therefore, we hypothesized that epigenetic changes, including DNA methylation at the 4q35 D4Z4 array and stability of epigenetic repression of the *DUX4-fl* mRNA, between individuals could account, at least in part, for the wide variability in clinical presentation of FSHD and similarly for the large number of asymptomatic individuals that fit the genetic criteria for FSHD1 [1,12,15,17,60,75,77]. Supporting this hypothesis, we found that myogenic cells from these FSHD1-nonmanifesting subjects have an intermediate epigenetic status at the pathogenic distal 4q35 D4Z4 repeat that is not as relaxed as that found in FSHD1 subjects manifesting weakness, but not as repressed as that found in healthy control subjects. In addition, DNA methylation levels at this region correlate with clinical disease, showing significant differences between the high methylation levels of healthy controls, the intermediate levels of FSHD1-nonmanifesting subjects, and the low levels of FSHD1-affected subjects. These differences in DNA methylation levels were significant in both a simple paired comparison between family members and also in a mixed-effect model including all samples (Figure 7).

This conclusion is in general agreement with a very recent publication that utilized the methyl-sensitive Southern blot method to investigate combined 4q and 10q D4Z4 DNA methylation levels at the proximal D4Z4 RU in FSHD1-affected and asymptomatic/nonpenetrant (comparable to our nonmanifesting) individuals [25]. The authors found that for those genetically FSHD1 subjects carrying 7 to 10 RUs at their shortest FSHD-permissive allele, affected subjects have significantly less DNA methylation than predicted based on their 4q and 10q D4Z4 array sizes, while asymptomatic subjects do not. This was interpreted as suggesting that for 7 to 10 RUs, additional factors beyond array size are likely involved in determining methylation levels, and clinical severity, for those with borderline contracted alleles [25]. This is in agreement with our finding that DNA methylation levels on the contracted allele for nonmanifesting subjects differ significantly from those for FSHD1-affected and healthy controls, representing an intermediate level of DNA methylation and epigenetic stability.

In light of this, there are several distinguishing features of our study. We show that in FSHD1 subjects, DNA methylation levels are altered specifically at the contracted distal 4qA D4Z4 RU, and these alterations correlate with disease severity. Importantly, our study goes beyond assaying CpG methylation levels in these subjects and shows that differential DNA methylation is functionally relevant, correlating with general epigenetic repression or relaxation of the contracted 4q35 D4Z4 array, as assayed by the expression of *DUX4-fl*. Myogenic cells from FSHD1-nonmanifesting subjects, which have intermediate DNA methylation at the distal 4q35 D4Z4 RU of the contracted allele, exhibit greater repression of *DUX4-fl* than cells from FSHD1-affected subjects, but less repression than healthy control cells. Interestingly, there is also variability in epigenetic repression among FSHD1-affected cells isolated from different subjects, suggesting that an individual’s epigenetic status may be an important aspect of clinical progression as well as disease presentation.

Considering that stable pathogenic *DUX4-fl* expression originates in the distal D4Z4 RU and extends to the permissive A-type subtelomere, it stands to reason that the distal unit on the contracted 4qA allele is the most critical region to analyze. However, due to technical limitations, all previous FSHD epigenetic studies had focused either on the proximal, non-pathogenic 4q/10q D4Z4 RU or on the random analysis of all 4q/10q D4Z4 RUs [4,20,25,50,51,76]. Our findings for this distal unit confirm earlier reports that hypomethylation in FSHD1 is restricted to the contracted 4q allele in subjects disomic for chromosome 4 type D4Z4 arrays [4] and moreover offer improved resolution of the allele-specific DNA methylation in two ways: first, in case of 4qA/4qA-L genotypes, the methylation of the two alleles is measured independently; second, for 4qA/4qA genotypes the measurement of methylation at multiple CpG sites per molecule allows us to estimate average methylation for each allele separately, rather than simply measuring the average methylation for both alleles combined.

The epigenetic status of the 4q35 distal D4Z4 region, as assayed by CpG methylation and *DUX4-fl* mRNA induction in response to epigenetic drugs, not only differs strongly between FSHD1-affected subjects and healthy controls, and between FSHD1-nonmanifesting subjects and healthy controls, but also differs between FSHD1-affected and FSHD1-nonmanifesting subjects within families (Figures 7 and 9). In fact, DNA methylation analysis of the distal 4qA D4Z4 RU could be used effectively as an FSHD biomarker that distinguishes healthy subjects from FSHD1-affected or FSHD1-nonmanifesting subjects. Within families, analysis of DNA methylation alone can generally distinguish between FSHD-affected and FSHD-nonmanifesting relatives (Table 2; cohorts 15, 28, 29, 30, 46, 47, 48, and 49); however, the differences in methylation levels between these genetically FSHD1 groups, while significant at the population level, are smaller than the differences found between either of the groups and healthy controls (Figure 7; Additional file 1: Table S2). Occasional families in which differences between affected and nonmanifesting subjects are minimal (for example, cohort 43), and variability in methylation levels between families, suggest that epigenetic factors in addition to DNA methylation are involved in determining if a subject will be clinically affected or disease nonmanifesting. Still, from a diagnostic standpoint, when combined with a clinical evaluation, this DNA methylation analysis will clearly identify both FSHD1-affected and FSHD1-nonmanifesting subjects from healthy (or non-FSHD) controls; the presence or absence of clinical symptoms consistent with FSHD will differentiate the two hypomethylated groups.

The current diagnostic techniques for FSHD1 include pulsed-field gel electrophoresis (PFGE) and molecular combing [78,79]. These tests can be diagnostic for FSHD1 in a patient with clinical symptoms if a contraction of the 4q35 D4Z4 array is identified ranging between 1 and 10 D4Z4 RUs in *cis* with an A-type subtelomere [15]; however, many people with RUs in the higher range (7 to 10 D4Z4 RUs) do not show any clinical manifestation of disease [20,33]. Therefore, PFGE and molecular combing have much less prognostic value for patients possessing D4Z4 contractions at the high end of the FSHD1 range. However, the epigenetic status of the distal D4Z4 RU does correlate with clinical manifestation and thus may be of more prognostic value.

Our results contrast with a recent study by Gaillard *et al*. [51], in which D4Z4 DNA methylation levels at the 3′ end of D4Z4s (near our 4qA BSS assay) were reported to be unchanged between FSHD1-affected, asymptomatic, and control cells while DNA methylation changes at the D4Z4 5′ region (similar to our DUX4 5′ BSS assay) could at best only distinguish some FSHD1-affected cells from some unaffected cells, grouping FSHD1 asymptomatic and healthy subjects together. Surprisingly, the authors report D4Z4 DNA methylation levels for FSHD1-asymptomatic cells that were equivalent across the repeat to those found in healthy control cells [51]. This discrepancy between the two studies must be addressed, as it has significant implications for both the clinic, with respect to diagnostics and potentially genetic counseling, and the lab, with respect to understanding disease establishment and mechanism as well as the design of therapeutic approaches. We have identified several critical technical differences between these two studies that can reconcile the data. First, we utilized familial cohorts of FSHD1 subjects with or without disease manifestations who all have D4Z4 repeat arrays of 5 to 8.5 RU (Table 2); the asymptomatic subjects analyzed in the Gaillard *et al*. study had 7 to 10 RU, which is the typical described range for asymptomatic subjects [56,57,75,80]. In our analysis, these FSHD1-affected subjects were analyzed separately (Figure 7) from FSHD1-affected subjects without familial nonmanifesting subjects, which tend to have smaller contracted alleles with less DNA methylation that could skew the analysis [20]. Additionally, our methylation analysis and interpretation of the DUX4 gene body is based on the distal 4qA D4Z4 RU; thus, either 100% (4qA/B) or approximately 50% (4qA/A) of the assayed chromosomes are from the contracted 4qA array. Therefore, we have specifically analyzed the methylation status of multiple independent sequences from the FSHD1-associated D4Z4, which is important because in FSHD1 only the contracted 4q D4Z4 array shows epigenetic changes [76]. In contrast, the Gaillard *et al*. study combined all FSHD1-affected subjects, regardless of repeat size or familial relationship, which potentially skewed the average methylation for FSHD1-affected subjects to be lower than if only FSHD1-affected subjects with similar repeat sizes as their FSHD1-asymptomatic subjects were analyzed. In addition, the BSS assays utilized by Gaillard *et al*., similar to our DUX4 5′ assay, do not distinguish between 4q and 10q D4Z4s and are therefore dominated by D4Z4 sequences derived from the expanded 4q/10q D4Z4 arrays, with sizes averaging between 25 and 60 RUs and potentially reaching over 100 RUs each, leaving D4Z4s from the much smaller contracted FSHD1-associated 4q D4Z4 array (*n* ≤ 11) as a clear minority in, and potentially altogether absent from, the assayed population. Therefore, in the analysis of ten randomly amplified D4Z4s, the impact of sequences from contracted 4qA alleles on the overall average methylation is expected to be small, and likely within the range of normal variation for the other alleles; thus, their analysis has severely limited statistical power. A further complication involves the sequence variability of BSS amplicons. 4q and 10q D4Z4 repeats have very few sequence polymorphisms [49], data supported by both of our BSS assays, which both show >99.8% identity to the expected reference sequence (Figures 3, 4, 5, and 6; Additional file 1: Figures S1 and S2), and others [52]. The presence of numerous sequence polymorphisms affecting expected CpG dinucleotides in the Gaillard *et al*. BSS analysis strongly suggests that D4Z4s were amplified from non-4q/10q D4Z4 homologs [49]. Considering that these D4Z4 homologs are not associated with FSHD or epigenetically altered in the disease [49], any inclusion of these sequences further complicates the methylation analysis, as it further dilutes the signal from the contracted 4qA allele (important for FSHD1) and also dilutes the signal from combined 4q/10q alleles (important for FSHD2). Thus, the discrepancy between our study and the Gaillard *et al.* study is likely due to differences in 1) class of subjects analyzed, 2) specificity of the BSS assays, and 3) statistical power of the analysis. It could be suggested that differences might result from our analysis being performed on fewer subjects; however, the fact that the smaller number of samples in our study produced much clearer and more significant differences actually highlights the power of our technique.

Overall, the DNA methylation results produced by our analysis are consistent with the majority of published literature for FSHD1-affected subjects and healthy controls, and the sequences analyzed are clearly specific for the FSHD1-associated D4Z4 array. Therefore, we conclude that FSHD1-nonmanifesting subjects have an intermediate DNA methylation state at the distal D4Z4 on the contracted 4qA allele that distinguishes them from FSHD1 subjects with muscle weakness and from healthy control subjects. In addition, this intermediate state is functionally relevant in that myocytes from FSHD1-nonmanifesting subjects exhibit more stable epigenetic repression than their counterparts from FSHD1-affected first-degree relatives. These different epigenetic states of the distal 4qA D4Z4 repeat can be used effectively as disease biomarkers that clearly distinguish between FSHD1 subjects and healthy controls regardless of any familial relation [48], have clinical implications for FSHD diagnostics and therapy development, and provide a basis for understanding the mechanism of disease establishment. For example, our results suggest that restoring even an intermediate level of DNA methylation or small increases in heterochromatinization of the D4Z4 array might be sufficient to lower *DUX4-fl* expression to a non-pathogenic level. In addition, DNA methylation has been found to decrease with age, and these aging-related changes are not global within a cell; some genomic regions change while others do not, and the changes are tissue-specific [81-83]. It is not known if the 4q35 D4Z4 array is susceptible to age-related changes in DNA methylation, but it is possible that the initial epigenetic status of contracted D4Z4 arrays could affect age-related demethylation and thus age of onset or severity of disease.

### FSHD1-sized D4Z4 arrays have characteristics of metastable epialleles

The epigenome consists of DNA methylation, histone post-translational modifications, and histone variants throughout the genome that together form an integral component of gene regulatory mechanisms [84-86]. Initially established during development, the epigenome organizes chromatin to restrict or facilitate the access of regulatory factors to DNA. Epigenetic marks provide a mechanism for regulatory memory that is passed on to subsequent cellular generations and is vital for maintaining cell-type specific patterns of expression and repression. The epigenetic modifications at the 4q35 D4Z4 array are established during early development [30] and differ among individuals. Potentially, variable aspects of the contracted D4Z4 array such as size or inherited DNA methylation patterns, when combined with an individual’s expression level or functional status of chromatin-modifying proteins such as SMCHD1, could shift the establishment of D4Z4 epigenetic repression in either direction. Similarly, stress, nutrition, and exposure to other environmental factors during critical points in development could influence the overall epigenetic state at the D4Z4 arrays. Once established, the epigenetic state would persist and provide protection from or susceptibility to aberrant *DUX4-fl* expression in muscle.

In addition to the strong influence of epigenetic regulation, another important aspect of FSHD1 contracted D4Z4 regions is the variegated gene expression of *DUX4-fl* mRNA, as both traits are characteristic of metastable epialleles. Metastable epialleles (reviewed in [43,44]), as opposed to traditional alleles, have variable expressivity leading to phenotypic mosaicism between individuals, as well as variegated cellular expression leading to phenotypic mosaicism between cells. This variable expression is not due to genetic heterogeneity, but rather is dependent on the epigenetic state, which is established in a probabilistic manner during development and then maintained in subsequent cellular generations. FSHD presents clinically with great variability in age of onset, affected muscles, rate of progression, and ultimate severity, even within families and among monozygotic twins [87-91]. The variegated *DUX4-fl* expression patterns in FSHD1 myogenic cells and the variable clinical manifestation in genetically FSHD1 individuals appear consistent with the FSHD1-associated *DUX4* allele functioning as a metastable epiallele [8].

## Conclusions

**Figure 10:**
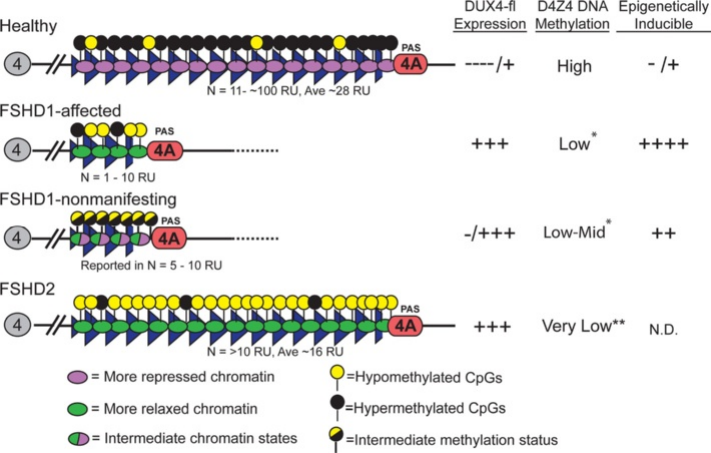
**FSHD1-affected, FSHD1-nonmanifesting, FSHD2, and healthy subjects are characterized by distinct states of epigenetic susceptibility to** ***DUX4-fl*** **expression.** Model for the different epigenetic states that distinguish healthy *vs.* FSHD1-affected *vs*. nonmanifesting *vs*. FSHD2 subjects. Healthy, unaffected subjects are characterized by stable repression of the distal pathogenic D4Z4 repeat, as indicated by DNA hypermethylation and chromatin compaction. Cells from these subjects express very low or undetectable levels of *DUX4-fl*, and are refractory to epigenetic induction of *DUX4-fl*. Cells from FSHD1-affected subjects display de-repression at the distal pathogenic D4Z4, as indicated by DNA hypomethylation and loss of chromatin compaction. These cells express detectable *DUX4-fl*, which is further induced upon treatment with epigenetic drugs. Cells from FSHD1-nonmanifesting subjects display an intermediate level of repression at the distal pathogenic D4Z4, as indicated by levels of DNA methylation and *DUX4-fl* inducibility which fall between those of FSHD1-affected and healthy, unaffected subjects. Despite lacking a contracted D4Z4 allele, cells from FSHD2 subjects are distinguished by severe hypomethylation at D4Z4 arrays, indicating a pronounced de-repression in these regions, which results in detectable expression of *DUX4-fl*. Interestingly, the shortest permissive 4q array in FSHD2 subjects tends to be shorter (approximately 16 RU) on average than the shortest 4q array in the healthy population (approximately 24 RU) [18,25]. *In genetically FSHD1 subjects, only the contracted 4q D4Z4 is hypomethylated; **In FSHD2 subjects, both 4q and 10q D4Z4 arrays are hypomethylated. Refer to text for more details.

FSHD is characterized by epigenetic dysregulation [8]. Here, we show that in the context of an FSHD1 disease-permissive allele, consisting of a contracted 4q D4Z4 in *cis* with a permissive A-type subtelomere, the epigenetic state of the 4q35 array is dominant over the genetic state in terms of disease outcome (Figure 10). Our DNA methylation analysis has uncovered distinct epigenetic states at the distal 4q D4Z4 array for unaffected, FSHD1-affected, and FSHD1-nonmanifesting subjects and has the potential to be used for diagnostic purposes. These different epigenetic states affect the stability of gene repression and potentially the splicing of the pathogenic *DUX4-fl* isoform. In addition, the contracted 4qA allele in genetically FSHD1 subjects has the characteristics of a metastable epiallele, which may impact disease establishment and progression, and provide an avenue to therapy via epigenetic manipulation.

## Methods

### Human subjects

This study was approved by the Johns Hopkins School of Medicine Institutional Review Board. Families with a member diagnosed with FSHD1 were invited to participate. Individuals were genotyped and considered to be affected with FSHD1 if a 4qA *Eco*RI/*Bln*I fragment <35 kb was identified using genomic DNAs isolated from peripheral blood mononuclear cells (PBMC) or considered to be healthy controls if they lacked a contracted 4qA allele (Additional file 1: Table S1). Haplotypes for both 4q alleles were determined for all subjects, as described [17]. All FSHD1 individuals were examined by an experienced neuromuscular physician (KRW). FSHD1 individuals were further characterized as ‘manifesting’ disease (affected) if they had weakness in the distribution classic for FSHD (for example, face, shoulder girdle, and biceps) or ‘nonmanifesting’ if they had full strength in this distribution.

### Clinical samples

Myogenic cells derived from the biceps muscles of genetically FSHD1 subjects (03Abic, 07Abic, 09Abic, 12Abic, 17Abic, 15Abic, 15Bbic, 16Abic, 19Abic, 21Abic, 28Abic, 28Bbic, 29Abic, 29Bbic, 30Abic, and 30Bbic) and their healthy unaffected first-degree relatives (03Ubic, 07Ubic, 09Ubic, 12Ubic, 16Ubic, 17Ubic, 17Vbic, 19Ubic, and 21Ubic) were used in this study (as previously described, Homma *et al.* [45]). All cells were obtained from the Paul. D. Wellstone Muscular Dystrophy CRC for FSHD at the University of Massachusetts Medical School, Worcester, MA (http://www.umassmed.edu/wellstone/). Myogenic cells were selected by FACS for CD56 expression such that all cultures were >90% desmin-positive [33,45]. Myogenic cells were grown on gelatin-coated dishes in high serum growth medium for proliferation then switched to low serum differentiation medium to induce myotube formation [33,45]. As described [92], proliferation of primary cultures of human myogenic cells began to slow at 55 to 60 population doublings as cells neared replicative limits. Therefore, all cells were used at <30 population doublings, except where indicated in subculturing experiments when cultures were examined at up to 47 population doublings. For all subjects in cohorts 39, 41, 43, 46, 47, 48, 49, and 51, DNA methylation analysis was performed on genomic DNAs isolated from PBMCs collected under IRB-approved protocols at the appropriate institution.

### Serial subcultures

Myogenic cells were cultured in growth medium on gelatin-coated plates to approximately 80% confluence, at which time cells were counted to calculate population doublings and passaged at 1:10 dilution. At each passage, cells were cultured in parallel on one 100-mm plate and one gelatin-coated four-well chamber slide. The culture in each plate was used to maintain myoblasts in growth medium for additional passaging, whereas the culture in each chamber slide was used to generate differentiated myotubes, which were analyzed for DUX4-FL and MyHC expression after 4 days in differentiation medium.

### Drug treatment

Stock solutions of 100 mM 5-Aza-2′-deoxycytidine/Decitabine, (Sigma-Aldrich A3656, St. Louis, MO, USA) in DMSO, 5 mM Trichostatin A solution (TSA, Sigma-Aldrich T1952), and 10 mM chaetocin (Sigma-Aldrich C9492) in DMSO were stored at −20°C and diluted with PBS just before adding to the culture. To minimize culturing artifacts, low passage (<30 population doublings) myoblast cultures were used for all experiments and culture pairs for affected *vs.* healthy or affected *vs.* nonmanifesting were within 1 passage of each other in all instances. Myoblasts were seeded on collagen-coated plates at a cell density of 1.6 × 10^3^/cm^2^. Starting the following day, Decitabine (5 μM final concentration) was added daily for a total of 3 days. When used, TSA (200 nM final concentration) or chaetocin (50 nM final concentration) was added to the cells for the last 24 h prior to sampling.

### Immunostaining

Myogenic cell cultures were fixed and co-immunostained for DUX4-FL and myosin heavy chain (MyHC). DUX4-FL was detected with either P4H2 mouse mAb as described [33] or rabbit mAb E5-5 (Epitomics, Burlingame, CA, USA) as described [47]. MyHC isoforms were detected with either mouse mAb MF20 or mouse mAb F59 [93], which were obtained from the Developmental Studies Hybridoma Bank developed under the auspices of the NICHD and maintained by the University of Iowa, Department of Biology, Iowa City, IA, USA. Nuclei were stained with bisbenzimide. The number of DUX4-FL-positive nuclei was determined from manually scanning the entire culture area. The number of nuclei in MyHC-positive cells and the total number of nuclei was approximated for each cell strain by counting 10 random fields of known area at 10X magnification and extrapolating to the total area of the well. Nuclei of 60,000 to 150,000 were screened for each cell culture. Cultures were imaged with a Nikon E800 fluorescence microscope with Spot camera and software, version 4.6 (Diagnostic Instruments, Inc., Sterling Heights, MI, USA).

### BSS DNA methylation analysis

For all subjects in cohorts 03, 07, 09, 12, 15, 16, 17, 19, 21, 28, 29, and 30, DNA methylation analysis was performed on genomic DNAs isolated from myocytes. For all subjects in cohorts 39, 41, 43, 46, 47, 48, 49, and 51, DNA methylation analysis was performed on genomic DNAs isolated from PBMCs. DNA methylation of the 4qA and 4qA-L distal regions was analyzed using the 4qA BSS and 4qA-L BSS assays, as described [23,48]. BSS analysis of 59 CpGs in the DUX4 promoter region (DUX4 5′ BSS assay) of 4q and 10q D4Z4 repeats was performed using primers BSS167F: TTTTGGGTTGGGTGGAGATTTT and BSS1036R: AACACCRTACCRAACTTACACCCTT, then followed by nested PCR with BSS475F: TTAGGAGGGAGGGAGGGAGGTAG and BSS1036R using 10% of the first PCR product. PCR products were cloned into the pGEM-T Easy vector (Promega, Madison, WI, USA), sequenced, and analyzed using web-based analysis software BISMA (http://biochem.jacobs-university.de/BDPC/BISMA/) [94] with the default parameters.

### Allele-specific DNA methylation estimation

The percentage of methylated CpG sites in a region can vary between alleles and can also vary between cells for the same allele. To prevent high methylation on the non-contracted 4qA allele from masking or diluting the signal for reduced methylation on the contracted 4qA allele (a weakness with methods that only measure overall average methylation [20]), we wish to estimate methylation for just the allele with lower methylation. For the purpose of distinguishing FSHD1-affected subjects from healthy controls, we proposed a simple score, the lower quartile (Q1) of percent methylation of all sequenced clones [48]. If two alleles have non-overlapping ranges of methylation and are represented in roughly equal proportions, this will approximate the median for just the allele with lower methylation. But if two alleles have overlapping ranges of methylation, Q1 is biased toward underestimating the median for the allele with lower methylation. Likewise, akin to the extreme cases in which two alleles have identical distributions, Q1 will underestimate the median methylation in cases where only one allele is amplified by the PCR assay, for example, if the other allele is a 4B, 4A-L, or 4A166 haplotype, which may not be known in advance. To reduce this bias, here we use a parametric model-based method for estimating allele-specific methylation.

The distribution of counts of methylated CpG sites across clones is not satisfactorily modeled by a binomial distribution, as the observed variance is typically approximately four times greater than that of a binomial distribution with the same mean and *N* (where *N* is the number of CpG sites; *N* = 56 for the 4qA assay, and *N* = 30 for the 4qA-L assay) (Additional file 1: Figure S3). This overdispersion is not simply due to the presence of two alleles with different methylation probabilities, as it is also seen when restricting the analysis to samples for which all clones arise from a single 4qA allele (for example, 4qA/4qB genotypes). This overdispersion can also not be addressed by allowing site-specific methylation probabilities for each CpG site (as in [95]) since by a convexity argument the resulting Poisson binomial distribution has variance at most as large as a standard binomial distribution with the same mean and same *N*.

To account for the overdispersion, the number of methylated CpGs for each allele (*i* = 1, 2) was modeled as a beta binomial distribution, where each clone (indexed by *j*) from the allele has an average methylation probability *p*_*ij*_ drawn independently from a beta distribution with parameters *a*_*i*_ and *b*_*i*_, and the observed number of methylated CpGs follows a binomial distribution with probability *p*_*ij*_ and sample size *N*. This distribution has the expected average CpG methylation fraction *a*_*i*_/(*a*_*i*_ + *b*_*i*_), with variance decreasing as *a*_*i*_ + *b*_*i*_ increases, approaching a binomial distribution in the limit of large *a*_*i*_ + *b*_*i*_. A Bayesian two-component mixture model was used to infer the parameters of the beta binomial distributions for each of the alleles and to compute the posterior probability of each sequence having originated from each allele, based on the observed methylation data. Note that unlike refs [95,96] we model just the total count of methylated CpGs, and not site-specific methylation probabilities; we also differ in using full Bayesian inference rather than maximum likelihood estimation.

The beta binomials were re-parameterized by *r*_*i*_ = log(*a*_*i*_/*b*_*i*_) and *s*_*i*_ = *a*_*i*_ + *b*_*i*_ for *i* =1, 2. To break the symmetry between the two alleles and impose a labeling of alleles so that *r*_1_ ≤ *r*_2_ we use a *N*(*μ* = 0, *σ* = 2) prior for the average of *r*_1_ and *r*_2_, and a zero-inflated gamma(*k* = 1, *β* = 0.5) distribution as a prior for the difference *d* = *r*_1_ − *r*_2_ ≥ 0. The zero-inflation puts a 0.5 prior probability mass on the difference being exactly zero, so the model can be used for 4qA/4qA, 4qA/4qB, or unknown genotypes. One could also adjust the prior based on known genotype data, or use the posterior probability that *d* > 0 as a measure of evidence for allele-specific methylation. We use a gamma(*k* = 1, *β* = 0.025) prior for *s*_1_ and *s*_2_. A small fraction of sequences are missing methylation data at a small number (1 to 3) of sites; *N* was decreased accordingly for these sequences. Posterior means for the parameters of interest were computed using Markov Chain Monte Carlo (MCMC), with the Rjags (v3-14) interface to the JAGS (v3.3.0) sampler. We used 1,000 MCMC steps for burn-in, followed by 30,000 MCMC steps for inference; convergence was monitored with the Gelman-Rubin diagnostic (PFSR < 1.01) [97] based on three chains run in parallel.

Additional file 1: Figure S4 (top) shows an example (16Abic) in which clones clearly separate into two clusters with distinct methylation percentages, and the two components of the mixture correspond to these two clusters, while allowing for slight deviations from 50% of clones in each cluster; Additional file 1: Figure S4 (bottom) shows an example (17Ubic) in which the clones do not clearly separate into two clusters, and the two estimated mixture components are nearly the same, with the allele of origin ambiguous for all clones; as the genotype of this sample is 4qA/4qB, we do not expect to see evidence of allele-specific methylation here. Bayesian allele-specific estimates depend on the prior probability distributions specified, but we confirmed that the reported differences between groups remained significant for other choices of parameters for the priors (twofold increase or decrease for standard deviation *σ* of normal prior and rate parameters *β* for gamma priors).

### Comparisons of DNA methylation between disease classes

For comparisons of DUX4 gene body methylation between FSHD-affected, nonmanifesting, and control samples, we first used the procedure described above to estimate the average methylation percentage for the 4A allele with lowest average methylation. For FSHD1 samples, this is expected to be the contracted D4Z4 4A allele. We use the same procedure for control samples with no contracted alleles for uniformity. We likewise use this procedure for samples believed to have only one amplified 4A allele; in such cases, the two allele-specific methylation estimates are typically quite close (within a percent or two, although larger deviations did sometimes occur, particularly in blood, perhaps representing increased mixing of multiple cell lineages).

We used a linear mixed effect (LME) model to fit the values *y* = log(*a*/*b*) for each sample, with fixed effects for cell type (myocyte or blood) and disease class (FSHD-affected, nonmanifesting, or control), including interactions between them, and a random effect for family. We also included an additive fixed effect for assay type (4qA or 4qA-L), as these assess different CpG sites that may have different baseline methylation percentages; indeed, for the 4qA assay, there are variations in CpG methylation probabilities across the length of the sequence, with the central third of the CpG sites typically showing less methylation than the first third (Additional file 1: Figure S5). Because we had limited 4qA-L data, we did not attempt to model interactions between assay type and cell type or disease class here. For sample 17A, which had both 4qA and 4qA-L alleles, we used the 4qA assay as it gave a smaller value of *y.* This corresponded to the shorter allele (19 kb *vs.* 87 kb) as desired; however, in the absence of genotyping data, a known baseline difference in methylation between 4qA and 4qA-L alleles could be adjusted for in deciding which should be regarded as the less methylated allele.

Note that *y* is equal to the log odds ratio log(*p*/(1 − *p*)), where *p* is the average fraction of CpG sites methylated. This logit transformation avoids the compression of values near *p* = 0 and *p* = 1. Estimated means and confidence intervals were transformed back to percentages in figures and tables. Models were fit using the R package lme4 (v1.1-7), and likelihood ratio tests were used for assessing significance. Because FSHD-affected subjects with nonmanifesting first-degree relatives may as a group differ from other FSHD subjects (due, for example, to nonmanifesting individuals tending to have borderline D4Z4 repeat lengths), we performed these tests with FSHD subjects divided into two subgroups, allowing nonmanifesting subjects to be compared with just their affected relatives (subgroup FSHD(b)) in a joint model that also includes the other FSHD cases (subgroup FSHD(a)) (for these particular FSHD samples, the two subgroups did not differ significantly; *P* = 0.29 by LRT). Likelihood ratios were computed between the full model and models with two of the four disease-call subgroups collapsed, or with the two cell types collapsed, with the lme4 function ‘anova’.

### qRT-PCR

Total RNAs were extracted using Trizol (Invitrogen, Carlsbad, CA, USA) and purified using the RNeasy Mini kit (Qiagen, Limburg, Netherlands) after on-column DNase I digestion. Total RNA (2 μg) was used for cDNA synthesis using Superscript III Reverse Transcriptase (Invitrogen), and 200 ng of cDNA were used for DUX4-fl qPCR analysis as described [33]. All data were normalized to levels of 18S rRNA [98]. Oligonucleotide primer sequences are provided in [33]. For the analysis of *ANKRD1* mRNA expression, 40 ng of cDNA were used with primers hANKRD1 For: GCCTACGTTTCTGAAGGCTG and Rev: GTGGATTCAAGCATATCACGGAA.

## Additional file

Additional file 1:
**Supplementary data.** **Table S1.** Characteristics of cell donors. **Table S2.** Summary of percent methylation. **Figure S1.** FSHD1-affected subjects are distinguished by lower levels of DNA methylation than healthy subjects at the 4q/10q D4Z4 5′ region. **Figure S2.** FSHD1-nonmanifesting subjects are distinguished by higher levels of DNA methylation than FSHD1-affected subjects at the 4q/10q D4Z4 5′ region. **Figure S3.** Within-sample variability in the number of methylated CpGs in the DUX4 gene body is greater than expected for a binomial distribution. **Figure S4.** Beta-binomial mixture model for the 4qA BSS assay. **Figure S5.** CpG methylation probabilities vary across the sequence in the 4qA BSS assay. **Figure S6.** Drug treatments have similar effects on control gene expression in FSHD1-affected and unaffected myocytes.

## Abbreviations

ADC: 5-aza-2′-deoxycytidine (Decitabine)
BS PCR: bisulfite PCR
BSS: bisulfite sequencing
CH: chaetocin
FSHD: facioscapulohumeral muscular dystrophy
LRT: likelihood ratio test
MyHC: myosin heavy chain
PCR: polymerase chain reaction
qRT-PCR: quantitative reverse transcriptase PCR
RU: repeat unit
TSA: Trichostatin A
