# Supplementary material for: Individual epigenetic status of the pathogenic D4Z4 macrosatellite correlates with disease in facioscapulohumeral muscular dystrophy
Source: Clin Epigenetics. 2015 Mar 29;7(1):37. doi: 10.1186/s13148-015-0072-6 (PMC4405830; doi:10.1186/s13148-015-0072-6)
Supplement: Additional file 1: — Supplementary data. Table S1. Characteristics of cell donors. Table S2. Summary of percent methylation. Figure S1. FSHD1-affected subjects are distinguished by lower levels of DNA methylation than healthy subjects at the 4q/10q D4Z4 5′ region. Figure S2. FSHD1-nonmanifesting subjects are distinguished by higher levels of DNA methylation than FSHD1-affected subjects at the 4q/10q D4Z4 5′ region. Figure S3. Within-sample variability in the number of methylated CpGs in the DUX4 gene body is greater than expected for a binomial distribution. Figure S4. Beta-binomial mixture model for the 4qA BSS assay. Figure S5. CpG methylation probabilities vary across the sequence in the 4qA BSS assay. Figure S6. Drug treatments have similar effects on control gene expression in FSHD1-affected and unaffected myocytes. [file 13148_2015_72_MOESM1_ESM.pdf]

Table S1. Characteristics of cell donors [1, 2]

Family	Donor*	Cells	Clinical	Familial Relations	Gender	Age[#] (yrs)	EcoRI/BlnI sizes, RUs§ and chr 4 haplotypes
03	03A	Myocyte	FSHD1	Proband**	F	40	20kb [~5.5RU] (4A161); 57kb [~17RU] (4B163)^^
	03U	Myocyte	Healthy	Sister of 03A	F	42	157kb [~47RU] (4A161); 80kb [~24RU] (4B163)^^
07	07A	Myocyte	FSHD1	Proband**	F	18	29kb [~8RU] (4A161); 53kb [~15RU] (4A161)
	07U	Myocyte	Healthy	Mother of 07A	F	49	53kb [~15RU] (4A161); 34kb [~9.5RU] (4B163)^^
09	09A	Myocyte	FSHD1	Proband**	F	31	25kb [~7RU] (4A161); >112kb [>33RU] (4B168)^^
	09U	Myocyte	Healthy	Mother of 09A	F	57	>112kb [>33RU] (4A161); >112kb [>33RU] (4A166H)
12	12A	Myocyte	FSHD1	Proband**	F	22	18kb [~5RU] (4A161); 63kb [~18RU] (4A161)
	12U	Myocyte	Healthy	Sister of 12A	F	24	>112kb [>33RU] (4A-L161)^; >112kb [>33RU] (4B168)^^
15	15A	Myocyte	FSHD1	Proband**	M	66	28kb [~8RU] (4A161); >112kb [>33RU] (4B163)^^
	15B	Myocyte	Nonmanifesting	Brother of 15A	M	69	28kb [~8RU] (4A161); >112kb [>33RU] (4B163)^^
16	16A	Myocyte	FSHD1	Proband**	F	56	20kb [~5.5RU] (4A161); 97kb [~29RU] (4A161)
	16U	Myocyte	Healthy	Sister of 16A	F	60	97kb [~29RU] (4A161); 56kb [~16RU] (4B168)^^
17	17A	Myocyte	FSHD1	Proband**	M	23	19kb [~5RU] (4A161); 87kb [~26RU] (4A-L161)^
	17U	Myocyte	Healthy	Brother of 17A	M	21	>112kb [>33RU] (4A161); 97kb [~29RU] (4B163)^^
	17V	Myocyte	Healthy	Father of 17A	M	50	87kb [~26RU] (4A-L161)^; >112kb [>33RU] (4B163)^^
19	19A	Myocyte	FSHD1	Proband**	M	65	22kb [~6RU] (4A161); 157kb [~47RU] (4A161)
	19U	Myocyte	Healthy	Daughter of 19A	F	41	79kb [~23RU] (4A161); 157kb [~47RU] (4A161)
21	21A	Myocyte	FSHD1	Proband**	F	82	26kb [~7.5RU] (4A161); >145kb [>43RU] (4A-L161)^
	21U	Myocyte	Healthy	Daughter of 21A	F	48	142kb [~42RU] (4A-L161)^; 63kb [~18RU] (4B163)^^
28	28A	Myocyte	FSHD1	Proband**	M	44	29kb [~8RU] (4A161); 75kb [~22RU] (4A161)
	28B	Myocyte	Nonmanifesting	Father of 28A	M	68	29kb [~8RU] (4A161); 117kb [~35RU] (4A166H)
29	29A	Myocyte	FSHD1	Proband**	M	39	30kb [~8.5RU] (4A161); 160kb [~48RU] (4A166)^^
	29B	Myocyte	Nonmanifesting	Mother of 29A	F	70	30kb [~8.5RU] (4A161); >160kb [>48RU] (4A161H)
30	30A	Myocyte	FSHD1	Proband**	M	57	30kb [~8.5RU] (4A161); 137kb [~41RU] (4B168)^^
	30B	Myocyte	Nonmanifesting	Sister of 30A	F	59	30kb [~8.5RU] (4A161); 81kb [~24RU] (4B163)^^

39	39A	PBMC	FSHD1	Proband**	M	45	30kb [~8.5RU] (4A161); 107kb [~32RU] (4A161)
	39U	PBMC	Healthy	Mother of 39A	F	75	107kb [~32RU] (4A161); 47kb [~14RU] (4B163)^^
41	41A	PBMC	FSHD1	Proband**	F	34	14kb [~3.5RU] (4A161); 102kb [~30RU] (4A166)^^
	41U	PBMC	Healthy	Father of 41A	M	54	102kb [~30RU] (4A166)^^; 87kb [~26RU] (4B162)^^
43	43A	PBMC	FSHD1	Proband**	F	33	19kb [~5RU] (4A161); 48kb [~14RU] (4B163)^^
	43B	PBMC	Nonmanifesting	Mother of 43A	F	62	19kb [~5RU] (4A161); 47kb [~14RU] (4B163)^^
46	46A	PBMC	FSHD1	Proband**	F	54	22kb [~6RU] (4A161); 122kb [~36RU] (4A161)
	46B	PBMC	Nonmanifesting	Sister of 46A	F	53	22kb [~6RU] (4A161); 132kb [~39RU] (4A161)
47	47A	PBMC	FSHD1	Proband**	M	30	30kb [~8.5RU] (4A161); 77kb [~23RU] (4A166)^^
	47B	PBMC	Nonmanifesting	Mother of 47A	F	51	30kb [~8.5RU] (4A161); 102kb [~30RU] (4A166)^^
	47C	PBMC	Asymptomatic	Sister of 47A	F	25	30kb [~8.5RU] (4A161); 112kb [~33RU] (4B163)^^
48	48A	PBMC	Nonmanifesting	Proband**	F	52	21kb [~6RU] (4A161); 67kb [~20RU] (4B163)^^
	48B	PBMC	FSHD1	Son of 48A	M	20	21kb [~6RU] (4A161); 77kb [~23RU] (4B168)^^
	48C	PBMC	FSHD1	Son of 48A	M	19	21kb [~6RU] (4A161); 92kb [~27RU] (4B163)^^
49	49A	PBMC	Nonmanifesting	Proband**	M	46	22kb [~6RU] (4A161); 147kb [~44RU] (4B163)^^
	49C	PBMC	FSHD1	Brother of 49A	M	56	22kb [~6RU] (4A161); >145kb [>43RU] (4B168)^^
51	51U	PBMC	Healthy	Mother of 51A	F	39	>145kb [>43RU] (4A161); 72kb [~21RU] (4B163)^^
	51C	PBMC	FSHD1	Father of 51A	M	43	29kb [~8RU] (4A161); 52kb [~15RU] (4B168)^^
	51D	PBMC	FSHD1	Sister of 51C	F	48	29kb [~8RU] (4A161); 52kb [~15RU] (4B168)^^

* Donors were designated by cohort (family) number (e.g., 07, 09, or 17) followed by a letter A-D for the genetically FSHD1 subjects or a letter U-Z for the unaffected first-degree relative(s).

** FSHD1 was confirmed by a shortened 4q D4Z4 repeat array identified by an EcoRI/BlnI restriction fragment of <35kb coupled with a 4qA subtelomere allele [1, 2].

§ Estimated number of D4Z4 repeat units (RUs) calculated by (EcoRI/BlnI fragment kb – 2)/3.3 = RU, rounded to nearest half-integer when RU < 10 and nearest integer otherwise.

Age at time of enrollment in the study

^ These alleles designated as 4A haplotypes by southern blotting are 4A-L and are not amplified or analyzed by the 4qA BSS assay; the 4qA-L BSS assay is used for the analysis of these alleles.

^^These alleles are the nonpermissive chromosome 4 haplotypes (4A166, 4B162, 4B163, 4B168) and not amplified by the 4qA or 4qA-L BSS assays.

Nonmanifesting is defined in this study as the subject having no discernible weakness on clinical examination.

1. Homma S, Chen JC, Rahimov F, Beermann ML, Hanger K, Bibat GM, Wagner KR, Kunkel LM, Emerson CP, Jr., Miller JB: **A unique library of myogenic cells from facioscapulohumeral muscular dystrophy subjects and unaffected relatives: family, disease and cell function.** *Eur J Hum Genet* 2012, **20:**404-410.

2. Jones TI, Chen JC, Rahimov F, Homma S, Arashiro P, Beermann ML, King OD, Miller JB, Kunkel LM, Emerson CP, Jr., et al: **Facioscapulohumeral muscular dystrophy family studies of DUX4 expression: evidence for disease modifiers and a quantitative model of pathogenesis.** *Hum Mol Genet* 2012, **21:**4419-4430.

Table S2: Summary of percent methylation

Subject	Cells	BSS.assay	Clinical	num.seqs	mean	min	Q1	median	Q3	max	est.low	est.high	est.both
03A	Myocyte	4qA	FSHD1	10	5.7	1.8	1.8	4.5	7.1	14.3	5.8	7.0	6.3
03U	Myocyte	4qA	Healthy	10	72.7	58.9	67.9	70.5	80.4	83.9	71.5	73.2	72.5
07A	Myocyte	4qA	FSHD1	18	24.5	5.4	7.1	25.9	28.6	58.9	17.8	30.0	24.8
07U	Myocyte	4qA	Healthy	16	50.2	10.7	38.7	58.0	62.5	69.6	35.3	59.0	49.4
09A	Myocyte	4qA	FSHD1	10	6.4	0.0	3.6	6.2	8.9	12.5	6.6	7.7	7.0
09U	Myocyte	4qA	Healthy	9	72.0	64.3	65.6	75.0	75.0	78.6	71.0	72.2	71.7
12A	Myocyte	4qA	FSHD1	10	28.4	10.7	17.9	26.8	35.7	50.0	25.6	31.4	28.6
12U	Myocyte	4qA−L	Healthy	12	84.2	70.0	76.7	85.0	93.3	96.7	82.1	85.7	83.9
15A	Myocyte	4qA	FSHD1	10	16.1	7.1	8.9	14.3	19.6	37.5	15.2	17.6	16.5
15B	Myocyte	4qA	Nonmanif	10	29.1	7.1	17.9	25.9	37.5	67.9	25.4	32.5	29.7
16A	Myocyte	4qA	FSHD1	15	39.2	1.8	8.9	42.9	62.9	87.5	9.5	59.4	38.6
16U	Myocyte	4qA	Healthy	36	40.7	5.4	30.6	42.9	53.6	73.2	34.4	47.1	40.4
17A	Myocyte	4qA	FSHD1	20	12.8	1.8	4.5	13.4	19.6	26.8	9.2	16.2	12.9
17A	Myocyte	4qA−L	FSHD1	12	76.1	60.0	68.3	78.3	83.3	93.3	74.6	77.1	75.9
17U	Myocyte	4qA	Healthy	20	71.4	58.9	64.3	69.6	76.8	92.9	70.0	72.3	71.4
21U	Myocyte	4qA−L	Healthy	12	82.5	63.3	78.3	83.3	86.7	93.3	81.3	82.7	82.1
28A	Myocyte	4qA	FSHD1	10	22.3	3.6	7.1	26.8	32.1	39.3	14.6	28.2	22.4
28B	Myocyte	4qA	Nonmanif	10	33.6	16.1	21.4	25.9	51.8	51.8	25.2	44.4	33.8
29A	Myocyte	4qA	FSHD1	11	6.7	0.0	3.6	5.4	10.7	14.3	6.5	8.0	7.2
29B	Myocyte	4qA	Nonmanif	20	40.4	1.8	8.9	41.1	70.5	78.6	12.5	68.7	38.7
30A	Myocyte	4qA	FSHD1	11	14.1	1.8	5.4	7.1	21.0	46.4	10.6	17.7	14.7
30B	Myocyte	4qA	Nonmanif	11	34.9	5.4	27.2	35.7	44.6	53.6	32.6	37.3	34.8
32A	PBMC	4qA−L	FSHD1	17	62.5	30.0	55.0	63.3	73.5	83.3	60.4	64.6	62.4
32U	PBMC	4qA−L	Healthy	16	74.5	46.7	73.3	76.3	81.7	90.0	73.0	75.9	74.4
39A	PBMC	4qA	FSHD1	15	38.7	1.8	16.1	42.9	65.2	73.2	16.3	58.5	37.7
39U	PBMC	4qA	Healthy	10	71.8	58.9	62.5	72.3	76.8	91.1	70.2	72.6	71.7
41A	PBMC	4qA	FSHD1	13	7.7	0.0	1.8	3.6	8.9	42.9	5.9	9.8	8.5
43A	PBMC	4qA	FSHD1	10	18.8	1.8	5.4	21.4	23.2	42.9	14.2	23.0	19.0
43B	PBMC	4qA	Nonmanif	12	30.5	0.0	11.6	38.4	44.6	53.6	14.5	42.5	29.5
46A	PBMC	4qA	FSHD1	14	35.7	0.0	14.3	38.4	64.3	67.9	13.7	56.5	33.6
46B	PBMC	4qA	Nonmanif	14	42.4	1.8	16.1	42.0	66.1	80.4	27.6	57.9	41.6
47A	PBMC	4qA	FSHD1	13	17.6	0.0	5.4	17.9	30.4	39.3	9.3	25.8	17.7
47B	PBMC	4qA	Nonmanif	11	16.5	5.4	8.5	14.3	24.6	33.9	14.9	18.6	16.8
47C	PBMC	4qA	Asymptom	12	24.7	1.8	10.7	22.7	38.8	55.4	16.9	31.9	24.7
48A	PBMC	4qA	Nonmanif	12	31.1	3.6	8.9	31.2	55.4	58.9	11.7	50.8	30.8
48B	PBMC	4qA	FSHD1	13	14.1	0.0	3.1	12.5	21.9	42.9	7.3	21.0	14.6
48C	PBMC	4qA	FSHD1	10	10.5	1.8	1.8	3.6	26.8	26.8	4.9	19.9	11.2
49A	PBMC	4qA	Nonmanif	11	27.3	1.8	12.5	25.5	42.4	57.1	18.8	35.4	27.2
49C	PBMC	4qA	FSHD1	12	19.5	3.6	4.5	10.7	39.3	50.0	8.0	41.6	20.1
51C	PBMC	4qA	FSHD1	11	24.2	1.8	17.9	21.4	36.2	42.9	21.5	26.9	24.3
51D	PBMC	4qA	FSHD1	14	25.0	3.6	14.3	22.3	33.9	67.9	22.4	27.5	25.4
51U	PBMC	4qA	Healthy	16	72.5	41.1	67.9	72.3	86.6	87.5	68.7	76.7	72.3
03A	Myocyte	DUX4 5'	FSHD1	20	26.7	0.0	8.5	21.2	45.8	67.2	14.3	39.4	26.1
03U	Myocyte	DUX4 5'	Healthy	15	77.2	64.4	69.9	72.9	86.0	93.2	74.9	79.2	77.1
07A	Myocyte	DUX4 5'	FSHD1	18	50.5	1.7	44.1	54.2	66.1	76.3	41.2	55.8	48.1
07U	Myocyte	DUX4 5'	Healthy	18	44.7	5.1	16.9	48.7	67.8	79.7	22.9	63.7	43.4
09A	Myocyte	DUX4 5'	FSHD1	19	34.5	0.0	7.2	25.4	60.6	91.5	15.1	50.4	34.5
09U	Myocyte	DUX4 5'	Healthy	19	56.8	31.0	37.3	55.9	74.6	86.4	41.1	72.4	57.0
12A	Myocyte	DUX4 5'	FSHD1	19	43.7	6.8	27.5	44.1	61.0	78.0	30.6	54.8	43.0
12U	Myocyte	DUX4 5'	Healthy	17	66.5	37.3	55.9	67.2	79.2	86.4	62.1	71.2	66.4
15A	Myocyte	DUX4 5'	FSHD1	20	40.1	0.0	20.3	44.9	57.6	81.4	23.8	52.6	37.5
15B	Myocyte	DUX4 5'	Nonmanif	20	64.2	6.8	43.2	75.4	85.6	91.5	40.5	82.0	62.0
17A	Myocyte	DUX4 5'	FSHD1	19	56.3	3.4	36.9	66.1	74.2	86.4	39.3	69.5	54.6
17U	Myocyte	DUX4 5'	Healthy	18	72.0	22.0	62.7	78.0	84.7	89.8	67.0	77.2	71.2
28A	Myocyte	DUX4 5'	FSHD1	20	16.6	0.0	5.1	14.4	24.6	50.8	10.6	22.1	16.9
28B	Myocyte	DUX4 5'	Nonmanif	20	31.4	3.4	19.5	29.7	43.2	83.1	30.4	33.4	31.8
29A	Myocyte	DUX4 5'	FSHD1	20	39.3	1.7	5.9	40.7	66.9	91.5	8.9	63.0	37.7
29B	Myocyte	DUX4 5'	Nonmanif	20	54.4	0.0	36.4	59.3	72.9	88.1	46.0	61.5	52.6
30A	Myocyte	DUX4 5'	FSHD1	20	38.9	0.0	21.2	36.4	55.9	86.4	35.0	42.2	36.7
30B	Myocyte	DUX4 5'	Nonmanif	20	30.8	3.4	16.9	29.0	45.8	59.3	24.0	37.7	30.8

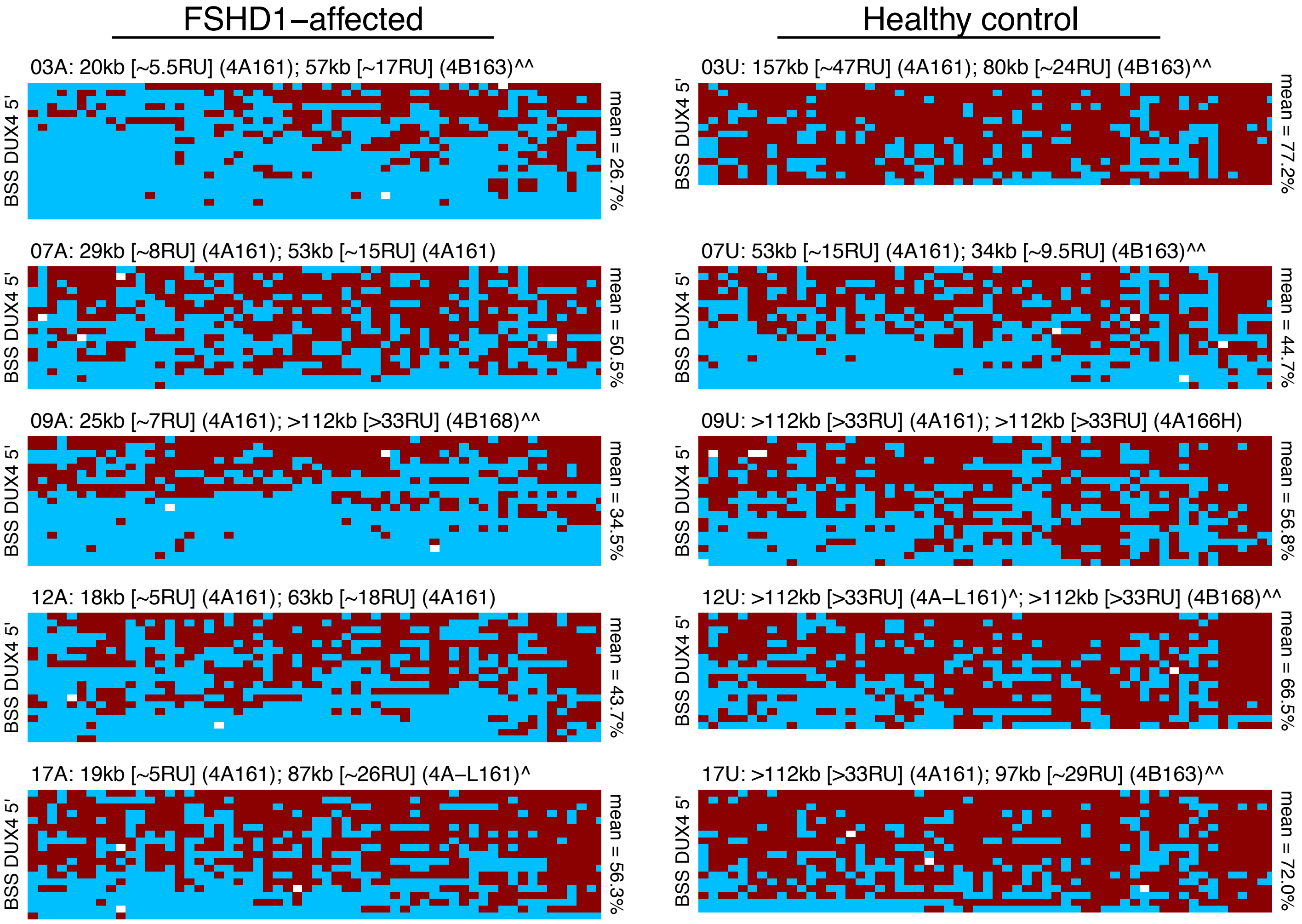

Figure S1: FSHD1-affected subjects are distinguished by lower levels of DNA methylation than healthy subjects at the 4q/10q D4Z4 5' region. BSS analysis (as described in Figure 3) of the D4Z4 5' region (Figure 2) in family cohorts of myogenic cells (03, 07, 09, 12, and 17) derived from biceps of FSHD1-affected (A) vs. healthy, unaffected subjects (U). Overall, 56 CpGs were assayed and ~95-100% of the predicted CpGs for the 4q/10q D4Z4 region were identified in all of the sequences analyzed, indicating that the amplified BSS reactions are specific to 4q and 10q D4Z4 repeats.

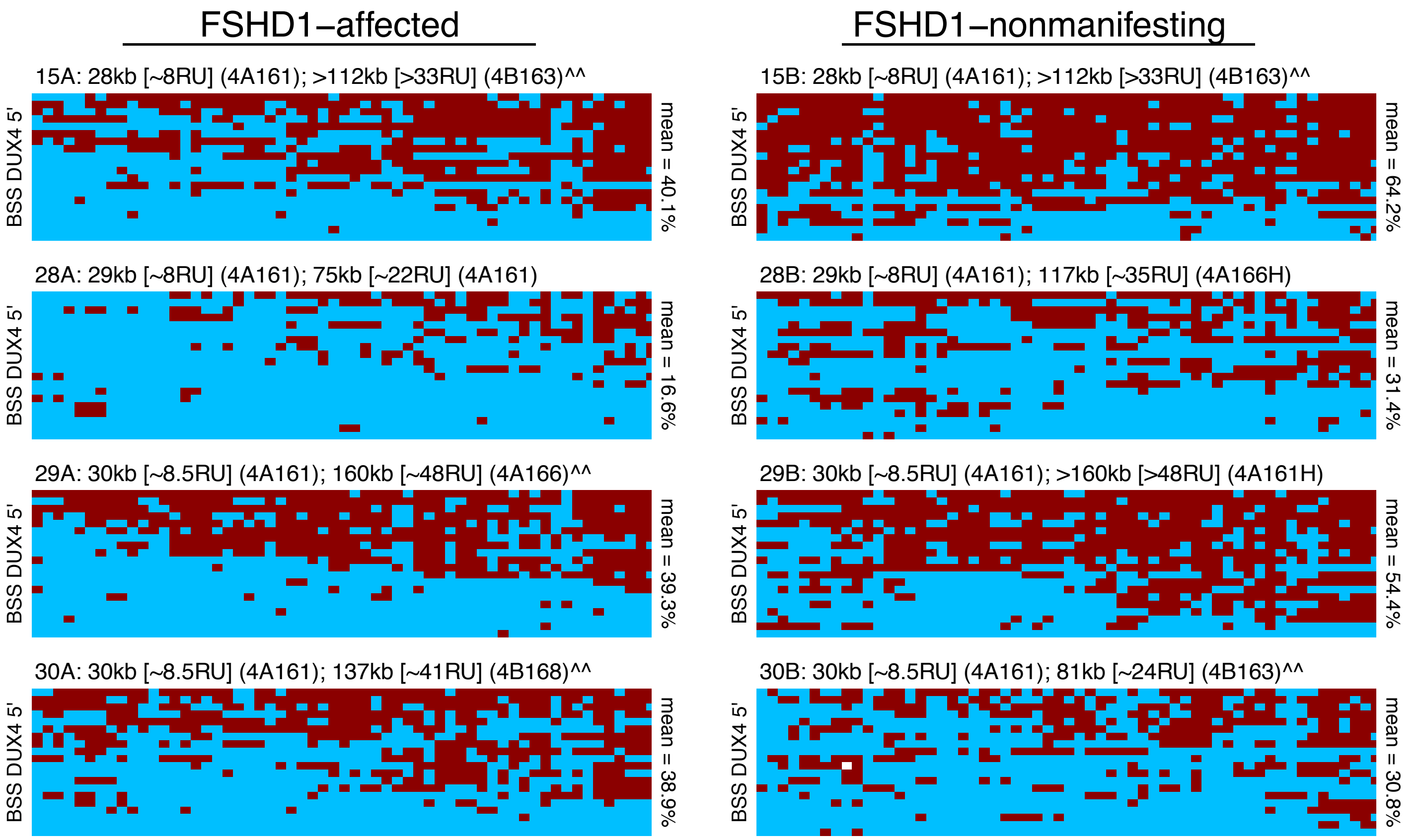

Figure S2: FSHD1-nonmanifesting subjects are distinguished by higher levels of DNA methylation than FSHD1-affected subjects at the 4q/10q D4Z4 5' region. BSS analysis (as described in Figure 3) of the D4Z4 5' region in family cohorts of myogenic cells (15, 28, 29, and 30) derived from biceps of FSHD1-affected (A) vs. FSHD1-nonmanifesting subjects (B). Overall, 56 CpGs were assayed and ~95-100% of the predicted CpGs for the 4q/10q D4Z4 region were identified in all of the sequences analyzed, indicating that the amplified BSS reactions are specific to 4q and 10q D4Z4 repeats.

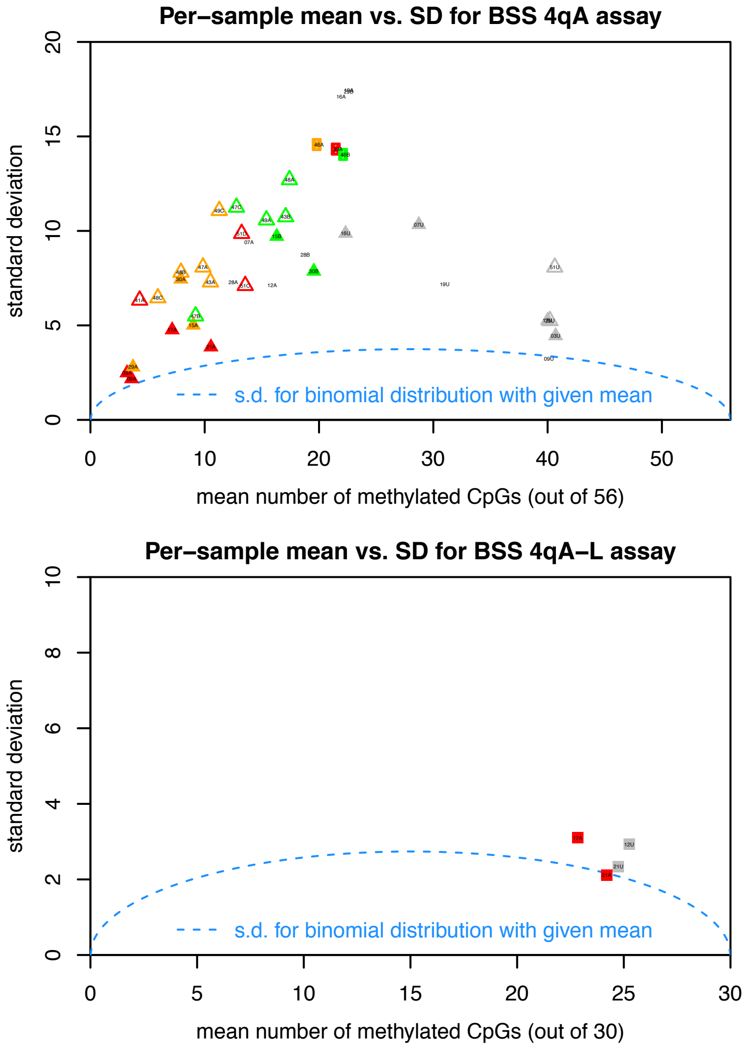

Figure S3: Within-sample variability in the number of methylated CpGs in the DUX4 gene body is greater than expected for a binomial distribution. The observed mean and standard deviation in the number of methylated CpGs, out of N = 56 total for 4qA assay (above) and N = 30 total for the 4qA-L assay (below), for different BSS sequences is shown for each sample. (For this figure the rare sequences with missing CpG data at one or more site were excluded.) Points are colored by disease group: orange for FSHD1 subjects with nonmanifesting relatives in the sample cohort, red for other FSHD1 subjects, green for non-manifesting subjects, grey for healthy controls. Solid symbols indicate myocyte samples, and empty symbols indicate blood samples. For the 4qA assay, triangles indicate genotypes with exactly one amplified 4qA allele (allele without ^ or ^^ in Table S1), and circles indicate samples that have more than one amplified 4qA; for the latter group, but not the former, part of the overdispersion may be attributed to methylation differences between alleles. For the 4qA-L assay, squares indicate genotypes with exactly on 4qA-L allele, which happens to account for all points. The standard deviation for a binomial distribution with given mean and N is indicated by the dashed blue line. The observed overdispersion relative to a binomial distribution, even for sample with a single amplified 4qA allele, motivates the use of a more flexible beta binomial distribution to model allele-specific methylation.

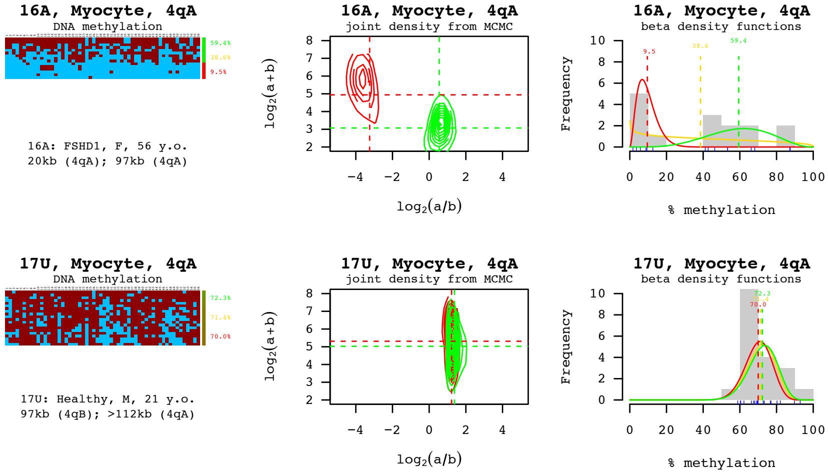

Figure S4: Beta-binomial mixture model for the 4qA BSS assay.

Figure S4: Beta-binomial mixture model for the 4qA BSS assay. Plots of beta-binomial mixture model of DNA methylation for 4qA BSS assay in myogenic cells from sample 16A **(top)** and 17U **(bottom)**. Each row has three panels: **(Left)** Grids show per-site CpG methylation for each bisulfite-sequenced clone (dark red = methylated; light blue = not methylated), with clones sorted from highest percent methylation to lowest. Numbers in the right margin indicate estimated percent methylation for each of two alleles using a beta-binomial mixture model (allele 1 in red, allele 2 in green), and using a single allele model (orange). The colorbar in the right margin indicates confidence in assignment of each sequence to each allele (a blend of red channel for posterior probability from allele 1 and green channel for posterior probability from allele 2). **(Center)** Contours of joint posterior probability density of $r_i = \log(a_i/b_i)$ and $\log(s_i) = \log(a_i + b_i)$ for $i = 1$ (red) and $i = 2$ (green) constructed from MCMC samples of mixture model. **(Right)** Histogram of observed methylation percentages for clones, with actual data points indicated by blue tick marks (jittered slightly to avoid overlap). Probability density functions for beta components of beta-binomial mixture model (using posterior mean estimates of r_i and s_i) are overlaid in red ($i = 1$) and green ($i = 2$); beta density for single allele beta-binomial model is shown in orange.

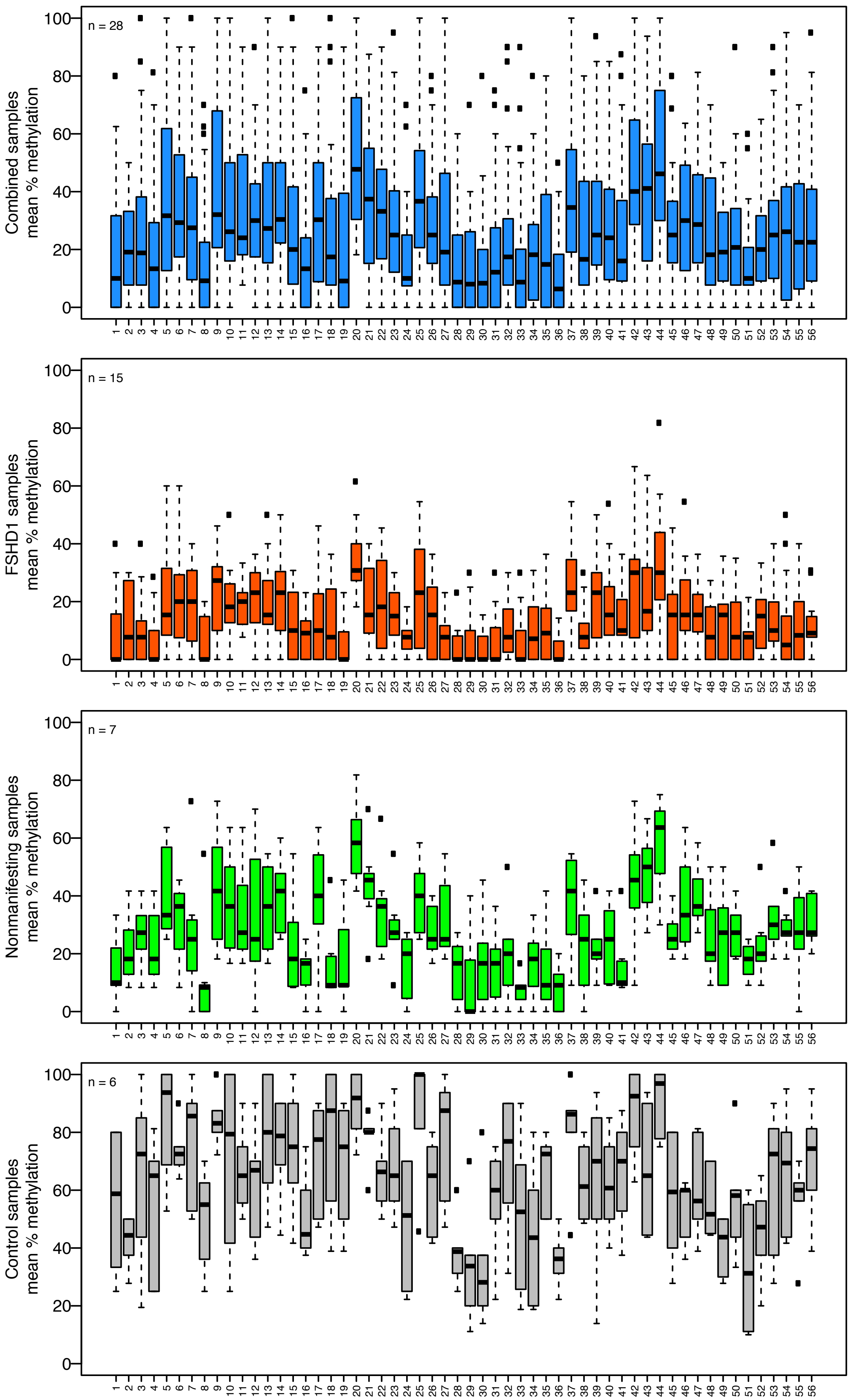

n = 28
Combined samples mean % methylation
n = 15
FSHD1 samples mean % methylation
n = 7
Nonmanifesting samples mean % methylation
n = 6
Control samples mean % methylation

Figure S5: CpG methylation probabilities vary across the sequence in the 4qA BSS assay. For each sample, the mean methylation percent at each of the 56 CpG sites was computed. These boxplots summarize the distribution of these per-site averages for different samples (bands indicate medians, and boxes extend from first quartile to third quartile). To sidestep complications due to mixtures of 4qA alleles, for these plots only those samples with a single amplified 4qA allele are included (alleles with ^ or ^^ symbol in Table S1 are not amplified). The upper plot (blue) shows 28 samples combined; the three other plots separate these samples into FSHD-affected (orange-red; 15 samples, combining what are elsewhere separated into FSHD(a) and FSHD(b)), nonmanifesting (green; 7 samples) and healthy control (grey; 6 samples).

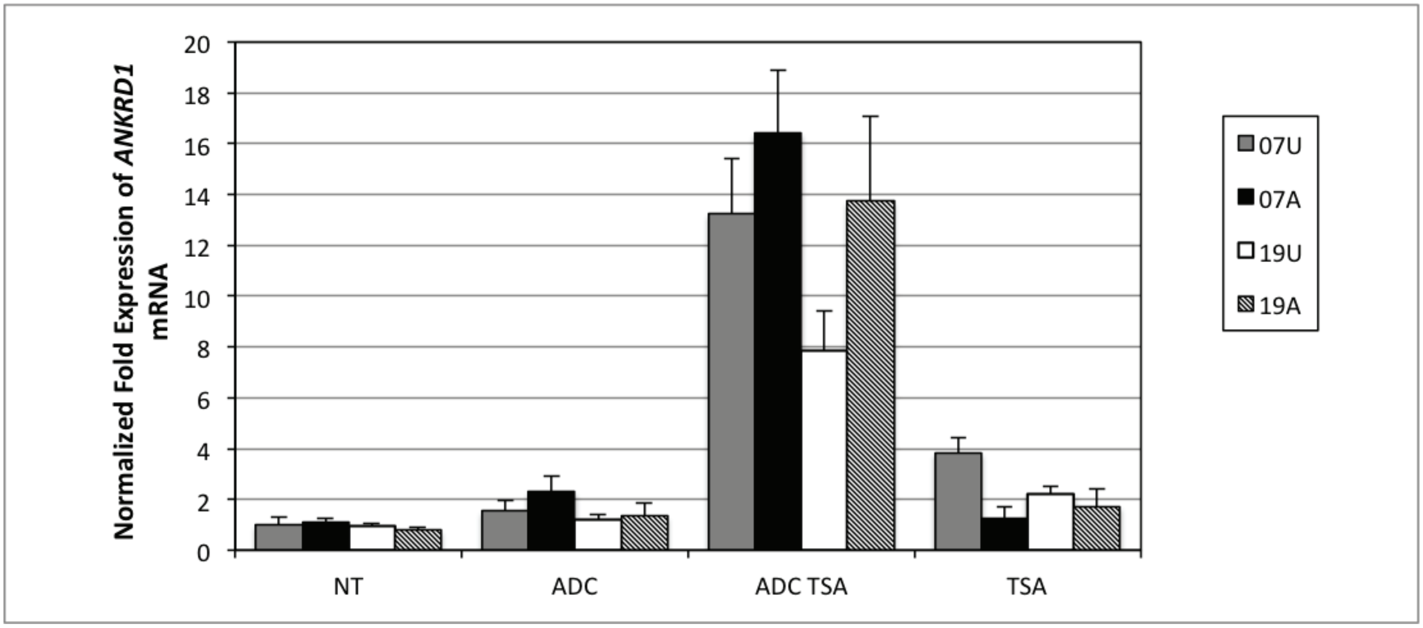

Figure S6: Drug treatments have similar effects on control gene expression in FSHD1-affected and unaffected myocytes. Two cohorts (07 and 19) that showed the greatest drug-induced *DUX4-fl* expression were assayed for expression of *Ankyrin Repeat Domain 1* (ANKRD1), a gene under epigenetic repression in myocytes. All four cell lines were similarly induced by each treatment with no significant differences between affected (A) and control (U) despite showing 20-fold and 32-fold induction for Decitabine (ADC) treatment and 7.5-fold and 65-fold induction for ADC TSA (cohort 07 and 19, respectively; Figure 8). Interestingly, the TSA alone treatment induced *ANKRD1* expression despite having no effect on *DUX4-fl* expression in these cohorts.
